# Comparative Transcriptomics Analysis of *Brassica napus* L. during Seed Maturation Reveals Dynamic Changes in Gene Expression between Embryos and Seed Coats and Distinct Expression Profiles of Acyl-CoA-Binding Proteins for Lipid Accumulation

**DOI:** 10.1093/pcp/pcz169

**Published:** 2019-08-28

**Authors:** Pan Liao, Helen K Woodfield, John L Harwood, Mee-Len Chye, Simon Scofield

**Affiliations:** 1 School of Biological Sciences, The University of Hong Kong, Pokfulam, Hong Kong, China; 2 School of Biosciences, Cardiff University, Cardiff CF10 3AX, UK

**Keywords:** ACBP, *Brassica napus*, Embryos, Fatty acid, Oilseed rape, Seed coats

## Abstract

Production of vegetable oils is a vital agricultural resource and oilseed rape (*Brassica napus*) is the third most important oil crop globally. Although the regulation of lipid biosynthesis in oilseeds is still not fully defined, the acyl-CoA-binding proteins (ACBPs) have been reported to be involved in such metabolism, including oil accumulation, in several plant species. In this study, progressive changes in gene expression in embryos and seed coats at different stages of seed development were comprehensively investigated by transcriptomic analyses in *B. napus*, revealing dynamic changes in the expression of genes involved in lipid biosynthesis. We show that genes encoding BnACBP proteins show distinct changes in expression at different developmental stages of seed development and show markedly different expression between embryos and seed coats. Both isoforms of the ankyrin-repeat *BnACBP2* increased during the oil accumulation period of embryo development. By contrast, the expression of the three most abundant isoforms of the small molecular mass *BnACBP6* in embryos showed progressive reduction, despite having the highest overall expression level. In seed coats, *BnACBP3*, *BnACBP4* and *BnACBP5* expression remained constant during development, whereas the two major isoforms of *BnACBP6* increased, contrasting with the data from embryos. We conclude that genes related to fatty acid and triacylglycerol biosynthesis showing dynamic expression changes may regulate the lipid distribution in embryos and seed coats of *B. napus* and that *BnACBP2* and *BnACBP6* are potentially important for oil accumulation.

**Accession numbers:** The transcriptome raw data are deposited at the Sequence Read Archive (SRA) database of GenBank under the accession number PRJNA510462. Sequence data for *BnACBPs* used in this study were retrieved from the CNS-Genoscope (http://www.genoscope.cns.fr/brassicanapus/; Chalhoub et�al. 2014): *BnACBP1* (BnaA02g10270D and BnaC02g44810D), *BnACBP2* (BnaA01g16660D and BnaC01g20440D), *BnACBP3* (BnaA01g13710D, BnaA03g46540D, BnaC01g16110D and BnaC07g38820D), *BnACBP4* (AIS76194, AIS76195, AIS76196, AIS76199, AIS76200 and AIS76201), *BnACBP5* (AIS76197 and AIS76199) and *BnACBP6* (BnaAnng25690D, BnaA05g36060D, BnaCnng15340D and BnaA08g07670D). *BnTIP41* (EV222761) was retrieved from the GenBank data library

## Introduction

Oil crops are a very important agricultural commodity contributing about 155 million tonnes of oil per annum (Gunstone et�al. 2007). Moreover, the demand for vegetable oils has been rising at 5% per year for the last 50 years (Gunstone et�al. 2007). With increased interest in plant oils as renewable chemicals and, to a lesser extent, as biofuels this demand is only likely to increase (Harwood et�al. 2017). Four major crops account for over 80% of total production and are, in order of importance, oil palm, soybean, rapeseed and sunflower (Weselake et al. 2017). Oilseed rape, mainly *Brassica napus* L., is the main oil crop in Canada and Northern Europe which contributes around 22% and 30% of the total rapeseed oil production, respectively (Carr� and Pouzet, 2014). Other *Brassica* species are grown in colder regions (*Brassica rapa*) or drier areas (*Brassica juncea*). Together oilseed rape accounts for around 16% of total vegetable oil production (Gunstone et�al. 2007, Taylor et�al. 2011). Oilseed rape is also the main source of biodiesel in Canada and Europe (Harwood et�al. 2017).

Triacylglycerol (TAG) is the dominant oil component and is produced in two overall steps. First, fatty acid (FA) biosynthesis in plastids is catalyzed by the multienzyme complexes of acetyl-CoA carboxylase and FA synthase. In most plants (and oilseed rape is typical), the main products of FA synthase, palmitate and stearate, are produced in a ratio of about 1:6. Stearate (as its acyl carrier protein ester) is a substrate for a very active Δ-9 desaturase in the chloroplast stroma to yield oleate. Apart from a few plants like cocoa (*Theobroma cacao*) or shea (*Butyrospermum parkii*), stearate rarely accumulates (Harwood et�al. 2017). The palmitate and oleate products of de novo FA formation are hydrolyzed by thioesterase enzymes (FATA and FATB; Salas and Ohlrogge 2002) and then re-esterified to coenzyme A by long-chain acyl-CoA synthetases (LACSs; Weselake et�al. 2009, Lu et�al. 2011, Bates et�al. 2013, Bates 2016). They then join the acyl-CoA pool in the cytosol. Acyl-CoA esters participate in various acyltransferase reactions for acyl lipid formation in the endoplasmic reticulum (ER). In addition, the production of phosphatidylcholine is important (Chen et�al. 2015) because this phosphoglyceride is a substrate for the ER FA desaturases (FAD2 and FAD3) that give rise to linoleate and α-linolenate, respectively (Wallis and Browse 2002).

It is clear that there is substantial movement of FAs from plastids to the ER during TAG accumulation in oil crops (Wallis and Browse 2002, Bates et�al. 2013). As lipids (even as thioesters) are poorly soluble in aqueous solutions, it is more efficient to bind them to proteins for transport. Moreover, acyl-CoAs are toxic to many enzymes through detergent-like effects and even at concentrations as low as 10^–9^ M can regulate metabolism (Faergeman and Knudsen 1997). Thus, binding (and transport) proteins have very important functions in vivo. Various proteins have been identified which can bind lipids (including acyl-CoAs; Du et�al. 2016) and these include acyl-CoA-binding proteins (ACBPs) of which there are six well-characterized forms in Arabidopsis and in rice (*Oryza sativa*; Du et�al. 2016).

The first ACBP reported in plants was a 10-kDa protein which was strongly expressed in seeds, flowers and cotyledons of oilseed rape (*B. napus* L.; Hills et�al. 1994). Further studies, especially in Arabidopsis and rice identified four different classes of ACBPs. Class I proteins are small molecular mass proteins (around 10 kDa) which are soluble and located in the cytosol, whereas Class II proteins have a transmembrane domain and ankyrin repeats. Class III ACBPs also have a transmembrane domain, whereas Class IV ACBPs have Kelch motifs but no transmembrane motif. All the ACBPs show a conserved acyl-CoA-binding domain (see Xiao and Chye 2011, Du et�al. 2016).

Apart from Arabidopsis and rice, ACBPs have been reported (and characterized) from a number of plants (Du et�al. 2016). Six ACBPs representing four classes are found in oilseed rape (Raboanatahiry et�al. 2015a, Raboanatahiry et�al. 2015b, Raboanatahiry et�al. 2018). The 10-kDa ACBP first reported by Hills et�al. (1994) was shown to bind various long-chain acyl-CoAs (Brown et�al. 1998, Yurchenko et�al. 2009). Notably, it also regulated the activities of important enzymes involved in oil synthesis such as glycerol 3-phosphate acyltransferase (GPAT; Brown et�al. 1998), lysophosphatidic acid acyltransferase (LPAAT; Brown et�al. 2002) and lysophosphatidylcholine acyltransferase (LPCAT; Yurchenko et�al. 2009). It was also shown to participate in acyl-CoA transport (Johnson et�al. 2002) and to promote exchange between the acyl-CoA and phosphoglyceride pools (Yurchenko et�al. 2009, Yurchenko et al. 2014). The function of ACBPs in FA biosynthesis has also been reported previously in Arabidopsis (Yurchenko et�al. 2009, Yurchenko et al. 2014, Lung et�al. 2017, Lung et al. 2018). The overexpression of *B. napus* 10-kDa ACBP in developing Arabidopsis seeds resulted in increased polyunsaturated FAs (18:2-FA and 18:3-FA), at the expense of saturated and very long monounsaturated FAs (20:1; Yurchenko et�al. 2009, Yurchenko et al. 2014). Furthermore, Arabidopsis ACBP1 overexpressors or an *acbp1* mutant showed changed FA composition in siliques and phloem, whereas an AtACBP3-RNAi line displayed a reduced FA content (Hu et�al. 2018).

Although the embryo is the major site for seed oil accumulation, the seed coat also plays an important role for lipid biosynthesis (Shi et�al. 2012, Woodfield et�al. 2017). The Arabidopsis transcription factor *GLABRA2* (*GL2*) regulates the *MUCILAGE MODIFIED4* (*MUM4*) gene in the seed coat which affects seed oil biosynthesis (Shi et�al. 2012). Moreover, loss of *GL2* or *MUM4* function in the seed coat resulted in increased seed oil, at the expense of seed coat mucilage biosynthesis (Shi et�al. 2012). The distinct lipid metabolism within seed coats compared with embryos (Woodfield et�al. 2017) allows a comparison in order to delineate the roles of individual proteins, such as ACBPs, in *B. napus*. Furthermore, although Arabidopsis ACBPs have been extensively studied regarding their function during stress (Xiao and Chye 2011), rather less is known of their role in development and oil accumulation in any plant species (Du et�al. 2016).

To understand the molecular basis of the different distribution of TAGs and phosphatidylcholines (PCs) between embryos and seed coats and to reveal putative ACBP functions for TAG biosynthesis during seed development, we conducted a comparative transcriptomics analysis and detailed study of *ACBP* expression during seed development in a low erucic acid (LEAR:Canola) variety of *B. napus*. The results show that genes related to FA biosynthesis in plastids may regulate the lipid distribution between embryos and seed coats. Also, the results reveal some very specific changes in expression patterns for different *BnACBPs* and their individual isoforms that may indicate not only distinct roles during oil accumulation and seed development but also tissue-specific differences in their functions.

## Results

### Characterization of *B. napus* seed morphology and oil content during development

**Fig. 1:**
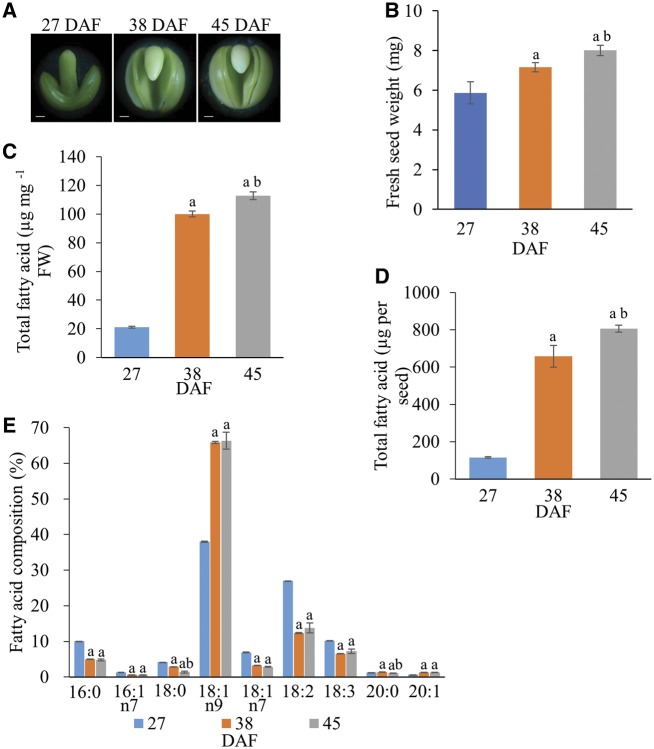
Analyses of morphology, fresh weight, total FA content and FA composition of seeds at three developmental stages. (A) Photos of embryos at 27, 38 and 45 DAF. Scale bar =200 �m. (B) Fresh seed weight at 27, 38 and 45 DAF. (C) Total FA content based on fresh weight at 27, 38 and 45 DAF. (D) Total FA content per seed at 27, 38 and 45 DAF. Seeds of wild-type *B. napus* cv. DH12075 (LEAR) were collected at 27, 38 and 45 DAF, representing early, rapid and late stages of oil accumulation in *B. napus*. FAs were analyzed by gas chromatography-mass spectrometry (GC-MS). Values are means � SD (*n* = 6); a, significant difference (*P *< 0.05 by Student’s *t*-test) when 38 or 45 DAF compared with 27 DAF; b, significant difference (*P *< 0.05 by Student’s *t*-test) between 38 and 45 DAF. Total FA was calculated based on fresh weight (FW) or of each fresh seed in relation to the internal control (19:0 FA). (E) FA composition of wild-type *B. napus* seeds at three developmental stages. Seeds of wild type were collected at 27, 38 and 45 DAF. Values are means � SD (*n* = 6); a, significant difference (*P *< 0.05 by Student’s *t*-test) when 38 or 45 DAF compared with 27 DAF; b, significant difference (*P *< 0.05 by Student’s *t*-test) between 38 and 45 DAF.

When the morphology of *B. napus* embryos at different developmental stages was analyzed, embryos at 27, 38 and 45 days after flowering (DAF; Fig.�1A) were identified to coincide with three important developmental stages that represent the early, rapid and late stages of oil accumulation in oilseed rape, respectively (Borisjuk et�al. 2013, Woodfield et�al. 2017, Woodfield et al. 2018). Fresh seed weight gradually increased throughout the period (**Fig. 1B**). FA content on a fresh weight basis increased from approximately 21 �g/mg at the early stage of oil accumulation (27 DAF) to approximately 100 �g/mg at 38 DAF. The latter time is toward the end of the rapid phase of oil accumulation (**Fig. 1C**) in agreement with the seed morphology (**Fig. 1A**; Borisjuk et�al. 2013). By 45 DAF, oil accumulation appeared to be complete (**Fig. 1C**), again in agreement with morphology (**Fig. 1A**). Values for total FAs per seed increased from 116 to 658 �g and then 806 �g/seed during the developmental period (**Fig. 1D**).

Because the development of oilseed rape was slower in Hong Kong than for other studies in Canada or Europe, we checked that the total FA composition of seeds was not affected significantly. During seed development from 27 to 45 DAF, there were notable decreases in the percentage of saturated (palmitate and stearate) and polyunsaturated FAs (linoleate and linolenate; **Fig. 1E**; Supplementary Table S1). These were compensated by the relative increase in oleate, as expected from the dominance of this acid in rapeseed oil. There was also a small increase in the percentage of eicosenoate and a decrease in that of *n*-7 octenoate (the latter noted to be concentrated in the seed coat of *B. napus*; Woodfield et�al. 2017). These data agreed with typical values for different low erucate cultivars of developing oilseed rape (Turnham and Northcote 1983, Gunstone et�al. 2007, Harwood et�al. 2017, Woodfield et�al. 2017). So, although the development of seeds was slower under our growth conditions, their overall characteristics (**Fig. 1****;**Supplementary Table S1) were as expected.

### Differential gene expression in embryos and seed coats during seed development

**Fig. 2:**
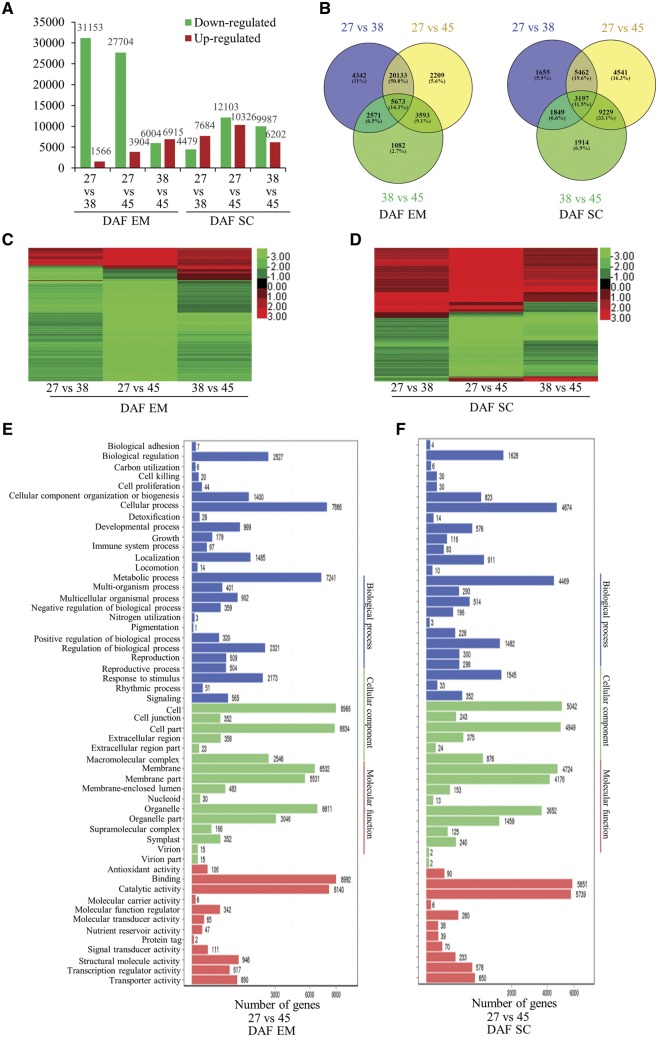
Identification of DEGs and cluster analysis of DEGs in *B. napus* embryos (EM) and seed coats (SC) during seed development. (A) Number of DEGs in embryos and seed coats at different developmental stages. DEGs were identified by the NoISeq method (see Materials and Methods section). FPKM was used for calculating expression levels. Definition of differential expression: (log_2_ ratio ≥1 and diverge probability ≥0.8). Red, upregulated genes; green, downregulated genes. (B) Venn diagram showing DEG overlaps among the 27 vs. 38 DAF, 27 vs. 45 DAF and 38 vs. 45 DAF pairwise comparisons in embryos (EM) and seed coats (SC). (C) Cluster analysis of DEGs in EM for each of the pairwise comparisons. Red, log_2_ ratio ≥0; green, log_2_ ratio ≤0. (D) Clustering as in (C) but for SC pairwise comparisons. (E) GO classification for DEGs identified in 27 vs. 45 DAF comparisons for EM. (F) GO classification for DEGs identified in 27 vs. 45 DAF comparisons for SC.

To understand the gene expression characteristics of each stage of seed development, we performed Next-Generation RNA-Sequencing (RNA-Seq) on *B. napus* embryos and seed coats at 27, 38 and 45 DAF, and subsequently performed a comparative transcriptomic analysis to identify genes that show differential expression throughout the different developmental stages. When comparative RNA-Seq analysis was performed on embryos and seed coats during seed development, the results indicated that 32,719, 31,608 and 12,919 differentially expressed genes (DEGs) were identified in embryos in 27 vs. 38 DAF, 27 vs. 45 DAF and 38 vs. 45 DAF comparisons, respectively (**Fig. 2A**), whereas 12,163, 22,429 and 16,189 DEGs were identified in the equivalent comparisons for seed coat samples (**Fig. 2A**). It was noted that the vast majority of DEGs identified in embryos (88–95%) was downregulated, in contrast to DEGs identified in seed coats (37–62%; **Fig. 2A–D**). We identified a core set of 5,673 genes that showed differential expression in all three time-point comparisons in embryos, and a set of 3,197 DEGs for equivalent comparisons in seed coat samples (**Fig. 2B**; Supplementary Table S2). In embryos, most DEGs in the core set showed downregulation throughout seed development (**Fig. 2C**), whereas in seed coats, the numbers of upregulated and downregulated DEGs were more equivalent (**Fig. 2D**).

Gene ontology (GO) category analysis showed that DEGs identified in both embryos and seed coats in the 27 vs. 45 DAF (i.e. earliest to latest developmental stage) comparison showed similar profiles for cellular component, with a high proportion of DEGs associated with ‘cell’, ‘cell part’, ‘organelle’, ‘membrane’ and ‘membrane part’ **(****Fig. 2****E, F)**. For the biological process category, DEGs in both embryos and seed coats were mostly associated with ‘cellular process’, ‘metabolic process’, ‘biological regulation’, ‘regulation of biological process’ and ‘response to stimulus’ **(****Fig. 2****E, F)**, whereas for the molecular function, ‘binding’ and ‘catalytic activity’ were the most highly represented categories (**Fig. 2E, F**). GO analysis of 27 vs. 45 DAF and 38 vs. 45 DAF yielded similar results (Supplementary Figs. S1, S2).

Pathway enrichment analysis was conducted by identifying the top 20 most enriched Kyoto Encyclopedia of Genes and Genomes (KEGG) pathways for DEGs from each comparison in embryo and seed coat tissue (Supplementary Figs. S3, S4). DEGs from both the embryo and seed coat samples for all three developmental stage comparisons were enriched for metabolic pathways, including enrichment of pathways associated with carbon fixation and metabolism, amino sugar and nucleotide sugar metabolism, photosynthesis, porphyrin and chlorophyll metabolism, and glyoxylate/dicarboxylate metabolism. FA biosynthesis/metabolism was also enriched in all comparisons and tissues except 27 vs. 38 DAF in seed coats, where enrichment of glycerolipid metabolism and biosynthesis of cutin, suberin and wax pathways was evident instead. Also in the 27 vs. 38 DAF seed coat comparison, we noted greater enrichment for pathways related to nucleic acid metabolism (including DNA replication and repair), whereas the equivalent embryo comparison showed greater enrichment of protein and amino acid metabolism pathways. It was also evident that the embryo samples showed greater enrichment of protein and amino acid metabolism pathways in the 27 vs. 45 DAF comparison, whereas the seed coat samples were more enriched for carbohydrate metabolism and the biosynthesis of secondary metabolites. In the 38 vs. 45 DAF comparisons, both embryos and seed coat samples showed enrichment of unsaturated FA biosynthesis and secondary metabolites. Similarly, KEGG second pathway term classification revealed that most DEGs mapped to translation, amino acid metabolism, carbohydrate metabolism, energy metabolism, lipid metabolism, transport and catabolism and folding, sorting and degradation (Supplementary Fig. S5).

### Expression dynamics of FA and lipid biosynthesis and storage genes during seed development

**Fig. 3:**
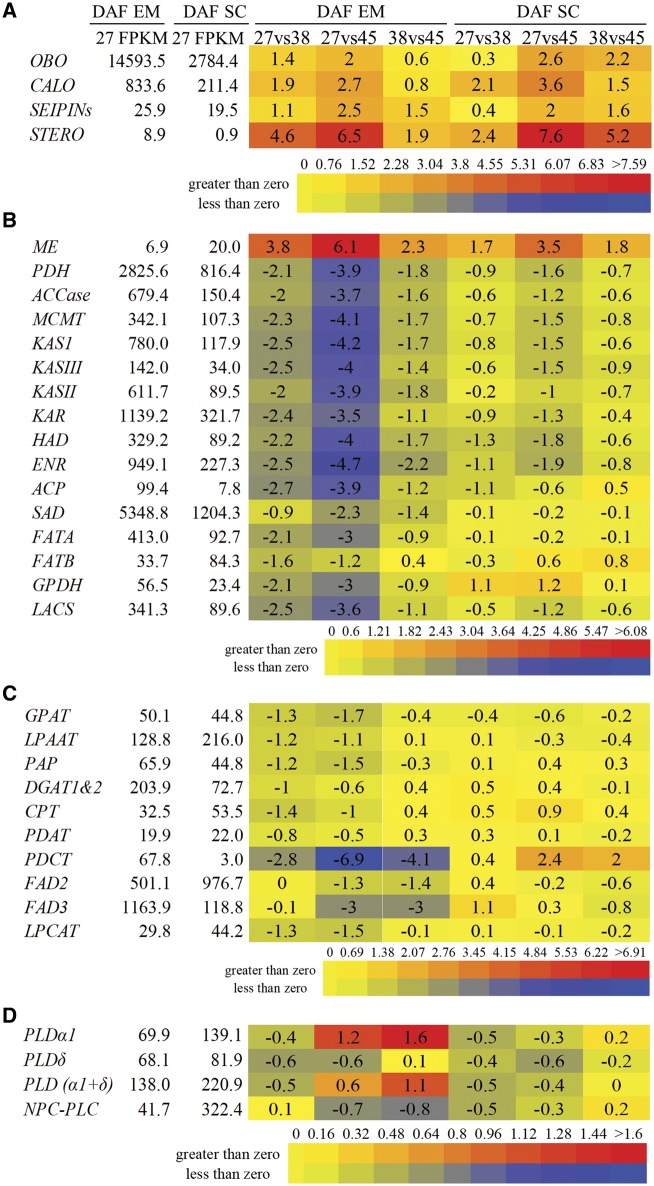
Differential expression of genes related to FA and lipid metabolism in *B. napus* embryos (EM) and seed coats (SC) during seed development. (A) Genes related to TAG storage, (B) FA biosynthesis in plastids, (C) TAG synthesis at the ER and (D) phospholipase C and D in *B. napus* embryos (EM) and seed coats (SC) during seed development. Average FPKM values from three biological repeats were used in calculating log_2_ ratio and diverge probability in each pairwise comparison. DEG: log2 ratio ≥1, diverge probability ≥0.8. Upregulated DEGs are marked in red and downregulated DEGs in blue. The color scale shows the magnitude of change in gene expression. Log_2_ ratio of FPKM in each sample pair was used in generating a heat map from http://bar.utoronto.ca/ntools/cgi-bin/ntools_heatmapper_plus.cgi. The *B. napus* genome reported by Chalhoub et al. (2014) was used as template for the identification of Gene ID in *B. napus*. ACCase, acetyl-CoA carboxylase; ACP, acyl carrier protein; CALO, caleosin; CPT, CDP-choline: diacylglycerol cholinephosphotransferase; DAF, days after flowering; DGAT, diacylglycerol acyltransferase; EM, embryo; ENR, enoyl-ACP reductase; FAD, fatty acid desaturase; FATA and FATB, fatty acyl-ACP thioesterases; FPKM, fragments per kilobase of transcript per million fragments mapped; GPAT, glycerol 3-phosphate acyltransferase; GPDH, glycerol-3-phosphate dehydrogenase; HAD, β-hydroxyacyl-ACP dehydratase; KAR, ketoacyl-ACP reductase; KAS, ketoacyl-ACP synthase; LACS, long-chain acyl-CoA synthetase; LPAAT, lysophosphatidic acid acyltransferase; LPCAT, lysophosphatidylcholine acyltransferase; ME, malic enzyme; MCMT, malonyl-CoA: ACP malonyltransferase; PLC, phospholipase C; OBO, oil body oleosin; PAP, phosphatidic acid phosphatase; PDH, pyruvate dehydrogenase; PDAT, phospholipid: diacylglycerol acyltransferase; PDCT, phosphatidylcholine: diacylglycerol cholinephosphotransferase; PLD, phospholipase D; SC, seed coat; SAD, stearoyl-ACP desaturases; SEIPINs, lipodystrophy proteins; STERO, steroleosins. For simplicity, expression levels of isoforms from a gene family have been combined where appropriate.

To further explore the possible mechanism that causes the differential distribution of TAGs and PCs between embryos and seed coats, expression analysis of genes related to TAG storage, FA biosynthesis in plastids, TAG synthesis in the ER and phospholipase C and D genes (Supplementary Fig. S6) was performed by RNA-Seq. Results indicated that the TAG storage-related genes were significantly increased during development (**Fig. 3A**), coinciding with the increased of lipid content during seed development (**Fig. 1C, D**). Most FA biosynthesis-related genes associated with plastids, including *PYRUVATE DEHYDROGENASE* (*PDH*), *ACETYL-COA CARBOXYLASE* (*ACCASE*), *MALONYL-COA: ACP MALONYLTRANSFERASE* (*MCMT*), *KETOACYL-ACP SYNTHASEI* (*KASI*), *KASIII*, *KASII*, *KETOACYL-ACP REDUCTASE* (*KAR*), *β-HYDROXYACYL-ACP DEHYDRATASE* (*HAD*), *ENOYL-ACP REDUCTASE* (*ENR*), *ACYL CARRIER PROTEIN* (*ACP*), *STEAROYL-ACP DESATURASE* (*SAD*), *FATA*, *GLYCEROL-3-PHOSPHATE DEHYDROGENASE* (*GPDH*) and *LACS* were significantly downregulated in embryos during seed development (**Fig. 3B**). Similarly, most of these genes showed downregulation in seed coats, though the magnitude of downregulation was reduced compared with embryos (**Fig. 3B**). It was noted that most FA biosynthesis-related genes were more highly expressed in 27 DAF embryos than seed coats, except *MALIC ENZYME* (*ME*) and *FATB* (**Fig. 3B**). ME catalyzes the conversion of malate to pyruvate, which can be used as precursors for the biosynthesis of FAs in plastids. Consistently, *ME* expression significantly increased in both embryos and seed coats during seed development (**Fig. 3B**). For genes related to TAG synthesis at ER, most were downregulated in embryos, including *GPAT*, *LPAAT*, *PHOSPHATIDIC ACID PHOSPHATASE* (*PAP*), *DIACYLGLYCEROL ACYLTRANSFERASES* (*DGAT1&2)*, *CDP-CHOLINE: DIACYLGLYCEROL CHOLINEPHOSPHOTRANSFERASE* (*CPT*), *PHOSPHOLIPID: DIACYLGLYCEROL ACYLTRANSFERASE* (*PDAT*), *PHOSPHATIDYLCHOLINE: DIACYLGLYCEROL CHOLINEPHOSPHOTRANSFERASE* (*PDCT*), *FAD2*, *FAD3* and *LPCAT* (**Fig. 3C**), while their expression levels were not notably reduced in seed coats (**Fig. 3C**). For phospholipase D and C genes, *PLDα1* was found to be upregulated in late-stage embryos and *NPC-PLC* showed a mild downregulation in 38 vs. 45 DAF, but no other significant differences were observed in either embryos or seed coats (**Fig. 3D**).

### Expression profiling of *BnACBPs* in embryos and seed coats during seed development

**Fig. 4:**
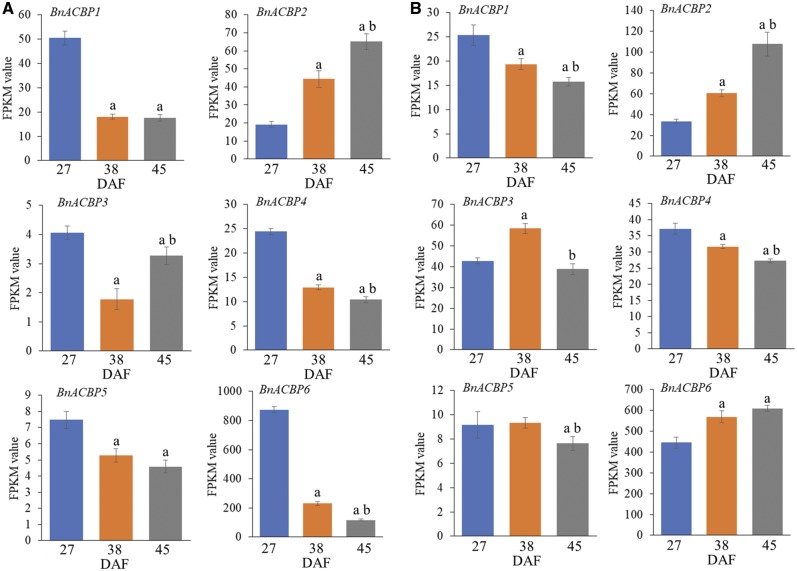
Expression profiling of *B. napus ACBPs* in embryos (A) and seed coats (B) during seed development by RNA-Seq. Total RNA was extracted from dissected embryos at 27, 38 and 45 DAF. Expression levels are represented by FPKM values. Data are means � SD of three independent replicates; a, significant difference (*P *< 0.05 by Student’s *t*-test) when 38 or 45 DAF compared with 27 DAF; b, significant difference (*P *< 0.05 by Student’s *t*-test) between 38 and 45 DAF.

Given their important role in oil accumulation **(**Supplementary Fig. S6), we analyzed the expression of genes encoding the ACBPs during seed development by RNA-Seq (**Fig. 4**) and validated results by quantitative real-time reverse transcription PCR (qRT-PCR; Supplementary Fig. S7). To obtain comprehensive information about the expression of *BnACBPs* in both embryos and seed coats and at the three different developmental stages (27, 38 and 45 DAF), RNA-Seq data were displayed in three different ways. First, the expression level (fragments per kilobase of transcript per million fragments mapped, FPKM) of the different *BnACBPs* in each tissue and the developmental stage was quantified (Supplementary Fig. S8). Second, changes in expression of each *BnACBP* were analyzed across the three developmental stages (**Fig. 4**; Supplementary Fig. S7). Third, the expression of each *BnACBP* was compared between embryos and seed coats (Supplementary Fig. S9).

In embryos, *BnACBP6* (encoding a small molecular mass Class I protein) was the most highly expressed of the *BnACBPs* throughout seed development (Supplementary Fig. S8), with the highest expression at the earliest developmental stage (**Fig. 4A**). At 27 DAF, *BnACBP1* (encoding a class II membrane-localized ankyrin-repeat protein) was the next highest expressed *ACBP*, followed by *BnACBP4* and *BnACBP2*, while *BnACBP3* and *BnACBP5* showed the lowest expression levels. When the expression of each *BnACBP* was compared at three different developmental stages, the results indicated that all *BnACBPs*, except *BnACBP2*, showed a decline during the period of rapid oil accumulation in embryo development, especially *BnACBP6* which showed the greatest relative reduction in expression level (**Fig. 4A**). Except for *BnACBP3*, this reduction was maintained or increased at maturity (45 DAF). By contrast, the expression of *BnACBP2* increased during seed development, demonstrating that this gene displays different expression dynamics to the other members of the *BnACBP* family. The expression profiles of *BnACBPs* in embryos during seed development were verified by qRT-PCR in independent experiments and the results were consistent with the RNA-Seq data: all *BnACBP* genes, except *BnACBP2*, showed declining expression during seed development, while *BnACBP2* expression increased (Supplementary Fig. S7A). These results independently confirm the RNA-Seq data.

In 27 DAF seed coats, *BnACBP6* was also the most highly expressed *ACBP*, while *BnACBP1*, *BnACBP2*, *BnACBP3* and *BnACBP4* showed similar, lower levels of expression and *BnACBP5* displaying the lowest expression level of all *BnACBPs* in this tissue (Supplementary Fig. S8). Changes in *ACBP* expression during seed development tended to be less dramatic in seed coats than in embryos. In contrast to embryos, *BnACBP6* expression in seed coats increased slightly throughout development (**Fig. 4B**) suggesting distinct roles for *BnACBP6* in the two tissues. *BnACBP2* expression increased substantially during development in seed coats, just as in embryos, whereas *BnACBP3* showed a transient increase at 38 DAF, suggesting that *BnACBP3* could play a role in seed coats during active oil accumulation. *BnACBP1* expression showed a mild decrease during seed development, as did *BnACBP4* and *BnACBP5* to a lesser degree. The expression profiles of *BnACBPs* in seed coats during seed development were validated by qRT-PCR **(**Supplementary Fig. S7B**)**, and showed an increase in *BnACBP2* and *BnACBP6* expression during seed development, with a transient peak of *BnACBP3* expression at the 38 DAF time point, whereas *BnACBP4* and *BnACBP5* showed a slight decrease in expression, consistent with the RNA-Seq data.

Direct comparison of the expression of *BnACBPs* in embryos and seed coats is shown in Supplementary Fig. S9. The data highlight the reciprocal expression dynamics of *BnACBP6*, with expression decreasing markedly in the embryo but increasing in the seed coat as seed development progressed. Conversely, *BnACBP2* showed increasing expression in both tissues during seed development. These differences in expression profile may reflect distinct roles of these *ACBPs* in embryos and seed coats. Furthermore, the differential expression of the Class I (ankyrin repeat) *ACBPs* (*BnACBP1* and *BnACBP2*) during seed development imply that, of these two genes, *BnACBP2* may have the predominant role in embryonic lipid accumulation, as it is most abundant ACBP of this class in the late-stage embryo (**Fig. 4****A;**Supplementary Fig. S8).

### Expression profiling of *BnACBP* isoforms in embryos and seed coats during seed development

*Brassica napus* ACBPs exist in a number of isoforms (Raboanatahiry et�al. 2015a) that arise from alternative splicing of pre-mRNAs transcribed from the six *BnACBP* loci. Two isoforms have been reported for *BnACBP1*, *BnACBP2* and *BnACBP5* and four isoforms for *BnACBP3*, *BnACBP4* and *BnACBP6* (Raboanatahiry et�al. 2015a, Raboanatahiry et al. 2015b). Table�1 shows the expression levels (FPKM) of the different *BnACBP* isoforms as determined by RNA-Seq. As noted above, *BnACBP6* shows the highest expression level in 27 DAF embryos, with *BnACBP1*, *BnACBP2* and *BnACBP4* the next highest expressed.

**Table 1:** Expression levels of *B. napus ACBP* isoforms in embryos and seed coats at three developmental stages by RNA-seq analysis

	27 DAF EM	38 DAF EM	45 DAF EM	27 DAF SC	38 DAF SC	45 DAF SC
BnACBP1-1	25.1 � 2.1	**9.5 � 0.8**	**7.5 � 1.1**	11.7 � 0.9	**9.6 � 1.6**	**7.0 � 0.6**
BnACBP1-2	25.3 � 3.6	**8.6 � 1.4**	**10.1 � 1.5**	13.7 � 3.4	**9.8 � 0.6**	**8.8 � 1.1**
BnACBP2-1	12.7 � 2.5	**29.1 � 7.6**	**37.2 � 6.9**	23.8 � 2.2	**46.0 � 3.6**	**75.9 � 15.4**
BnACBP2-2	6.5 � 0.8	**15.3 � 1.7**	**27.9 � 2.1**	9.7 � 1.5	**14.4 � 3.0**	**31.7 � 7.8**
BnACBP3-1	0.7 � 0.3	**0.2 � 0.2**	**0.1 � 0.1**	4.6 � 1.0	**7.0 � 1.0**	**7.1 � 1.5**
BnACBP3-2	1.3 � 0.4	0.9 � 0.8	**2.8 � 0.9**	14.7 � 0.5	**18.7 � 1.9**	**10.1 � 2.4**
BnACBP3-3	0.7 � 0.2	**0.3 � 0.1**	**0.1 � 0.0**	10.2 � 2.5	**13.9 � 2.9**	**12.6 � 3.2**
BnACBP3-4	1.4 � 0.1	**0.4 � 0.4**	**0.2 � 0.1**	13.3 � 1.6	18.8 � 3.8	**8.9 � 3.3**
BnACBP4-1	3.0 � 0.8	**2.0 � 0.3**	**1.7 � 0.4**	5.8 � 1.7	5.1 � 0.3	**4.0 � 0.4**
BnACBP4-2	4.0 � 0.1	**1.9 � 0.4**	**2.1 � 0.1**	6.0 � 1.7	5.1 � 0.6	**4.5 � 0.4**
BnACBP4-3	11.4 � 0.5	**6.4 � 0.8**	**3.4 � 0.9**	18.8 � 2.6	**14.2 � 1.0**	**11.2 � 1.1**
BnACBP4-4	6.0 � 0.9	**2.6 � 0.3**	**3.2 � 1.1**	6.6 � 0.8	7.3 � 0.6	7.5 � 0.7
BnACBP5-1	4.0 � 0.6	**2.7 � 0.3**	**2.2 � 0.4**	4.3 � 1.4	4.5 � 0.7	**3.2 � 0.3**
BnACBP5-2	3.4 � 0.5	**2.6 � 0.5**	**2.4 � 0.4**	4.9 � 0.8	4.8 � 0.2	4.5 � 0.8
BnACBP6-1	224.7 � 10.5	**50.2 � 16.4**	**13.0 � 7.3**	96.2 � 16.7	61.7 � 20.5	**37.6 � 4.1**
BnACBP6-2	435.4 � 35.4	**132.3 � 30.3**	**70.2 � 16.5**	202.4 � 57.2	**284.6 � 62.6**	**341.2 � 36.1**
BnACBP6-3	205.4 � 38.7	**40.3 � 6.3**	**21.8 � 4.9**	114.3 � 23.7	**184.4 � 26.3**	**190.9 � 4.5**
BnACBP6-4	8.6 � 1.0	7.5 � 0.4	12.5 � 2.6	32.7 � 9.1	38.4 � 7.4	40.0 � 9.4

Seeds were collected at 27, 38, and 45 days after flowering (DAF). Total RNA was extracted from dissected embryos at 27, 38, and 45 DAF. The expression levels of *B. napus ACBP* isoforms were represented by fragments per kilobase of transcript per million fragments mapped (FPKM) values generated from RNA-seq data. Data are means **�** SD of three independent replicates. Values displayed significant changes (*P* < 0.05 by Student’s *t*-test) are shown in bold. Values increased in comparison to control (27 DAF EM for embryos or 27 DAF SC for seed coats) are marked in red, values decreased in comparison to control (27 DAF EM for embryos or 27 DAF SC for seed coats) are marked in blue.

**Fig. 5:**
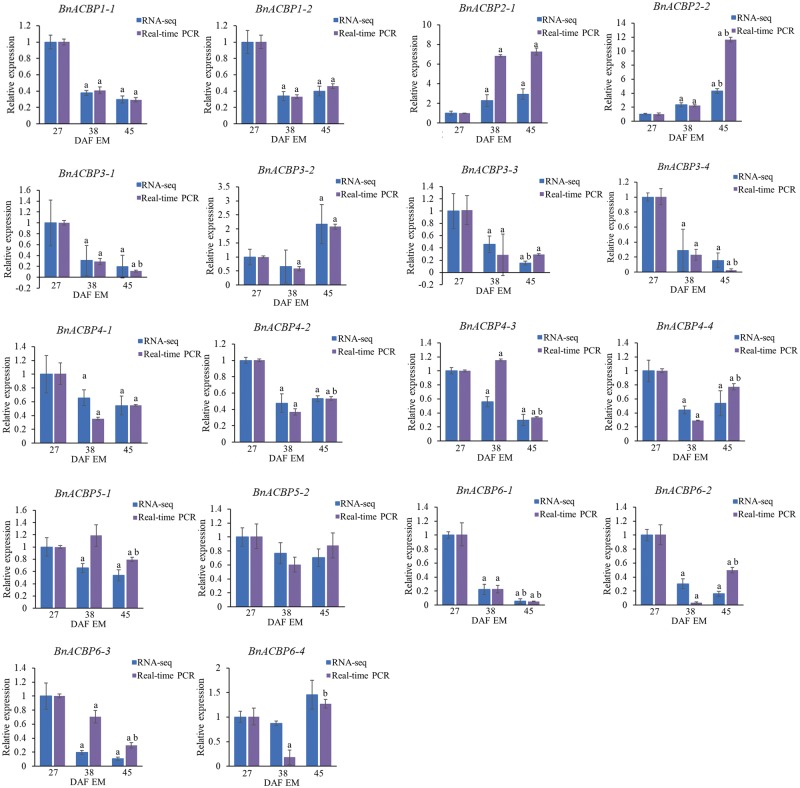
Expression profiling of individual *BnACBP* isoforms in embryos (EM) during seed development by RNA-Seq and qRT-PCR analyses. Gene expression levels were analyzed at 27, 38 and 45 DAF. For RNA-Seq data, expression levels represented by FPKM values were normalized to 27 DAF. For qRT-PCR analysis, 27 DAF sample was used as a baseline and expression of *TIP41* was used for normalization. Data are means � SD of three independent replicates; a, significant difference (*P *< 0.05 by Student’s *t*-test) when 38 or 45 DAF were compared with 27 DAF; b, significant difference (*P *< 0.05 by Student’s *t*-test) between 38 and 45 DAF.

**Fig. 6:**
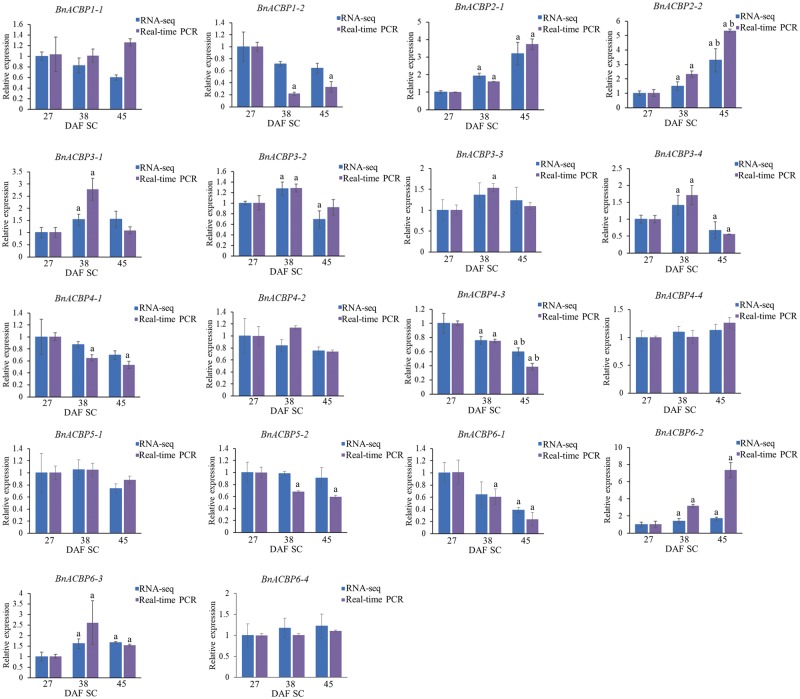
Expression profiling of individual *BnACBP* isoforms in seed coats (SC) during seed development by RNA-Seq and qRT-PCR analyses. Gene expression levels were analyzed at 27, 38 and 45 DAF. For RNA-Seq data, expression levels represented by FPKM values were normalized to 27 DAF. For qRT-PCR analysis, 27 DAF sample was used as a baseline and expression of *TIP41* was used for normalization. Data are means � SD of three independent replicates; a, significant difference (*P *< 0.05 by Student’s *t*-test) when 38 or 45 DAF were compared with 27 DAF; b, significant difference (*P *< 0.05 by Student’s *t*-test) between 38 and 45 DAF.

When the expression of individual *BnACBP* isoforms in embryos and seed coats was analyzed by RNA-Seq and qRT-PCR, the results (**Figs. 5, 6**) were largely consistent with the nonisoform-specific expression analysis described above. In embryos, the expression of both *BnACBP1* isoforms decreased at 38 and 45 DAF, whereas the *BnACBP2* isoforms increased significantly (**Fig. 5**). For *BnACBP3*, isoforms *3-1*, *3-3* and *3-4* all decreased but *BnACBP3-2* increased at the end of seed development. For *BnACBP4*, there was a general decline in all four isoforms while for the two *BnACBP5* isoforms, there were rather small decreases which were only significant for some comparisons (**Fig. 5**). *BnACBP6* isoforms *6-1*, *6-2* and *6-3* were substantially decreased during seed development, whereas *BnACBP6-4* did not show such a reduction at 45 DAF and was instead mildly increased (**Fig. 5**). Hence, the gene expression profiles for the different alternatively spliced *BnACBP* isoforms largely reflect those for the canonical *BnACBP* transcripts described earlier.

The expression of *BnACBP* isoforms in seed coats differed significantly from that in embryos during seed development. *BnACBP1-1* showed little change while *BnACBP1-2* was deceased (**Fig. 6**). By contrast, both isoforms of *BnACBP2* had increased expression during development. For *BnACBP3*, all four isoforms showed increased expression during the period of rapid oil accumulation (38 DAF) after which they declined to early-stage levels or slightly less (**Fig. 6**). For *BnACBP4*, isoforms *4-1* and *4-3* showed reduced expression during development while isoforms *4-2* and *4-3* showed little change in expression, as did both isoforms of *BnACBP5* (**Fig. 6**). The *BnACBP6-1* expression was reduced during development, whereas *BnACBP6-4* did not alter. Both *BnACBP6-2* and *BnACBP6-3* increased in expression during seed development (**Fig. 6**).

In summary, the expression profiles of the different *BnACBP* isoforms confirm the earlier observation that the expression patterns in seed coats and embryos are quite distinct at the different developmental stages, thus pointing to different temporal and tissue-specific functions for *ACBPs* in these two tissues during seed development. Furthermore, they also show differential patterns of expression for different isoforms of individual *ACBPs*, revealing an unexpected level of complexity in the patterns of *ACBP* isoform expression which is likely to be related to their individual functions.

## Discussion

### Dynamic changes in gene expression in embryos and seed coats during seed development

In this study, a comparative transcriptomics analysis was performed to understand the changes in gene expression that occur during the critical phase for oil accumulation in *B. napus* seed development, and to reveal important differences in gene expression between the embryo (in which most oil accumulates) and the seed coat. When comparing the gene expression profiles among different stages of seed development (27, 38 and 45 DAF), more DEGs were identified in embryos than seed coats. Although most DEGs were progressively downregulated in embryos during seed development, a smaller proportion of DEGs were downregulated in seed coats, indicating different gene expression dynamics in these two tissues during seed development.

During seed development, oil is synthesized and accumulates mainly in the embryo. Consistent with this, we observed larger expression changes in DEGs associated with FA and lipid biosynthesis and storage in embryos than in seed coats. Most DEGs in the embryo were downregulated during development, especially those related to FA and TAG biosynthesis, consistent with a previous study (Troncoso-Ponce et�al. 2011). However, genes related to oil storage increased in expression level during seed development, consistent with the progressive increase in oil content. Although most genes involved in FA biosynthesis in plastids were downregulated in both embryos and seed coats, some ER-associated TAG synthesis-related genes were reciprocally expressed between embryos and seed coats, suggesting that these differences may cause the distinct distribution of TAGs and PCs between these different tissues as previously observed (Woodfield et�al. 2017).

### ACBP expression and function during embryo development

*BnACBP6*, the only member of the small molecular mass Class-1 ACBPs, was the most highly expressed ACBP at all developmental stages, although its expression declined steadily during seed development. At 27 DAF, *BnACBP1* showed the next highest expression level, followed by *BnACBP4* and *BnACBP2*. Of these, the steady increase in expression of *BnACBP2*, which encodes a Class-2 ankyrin-repeat protein, is notable and may suggest its involvement in oil accumulation and, later, in seed maturation. *BnACBP3* and *BnACBP5* are expressed at very low levels and are, therefore, unlikely to be important for lipid formation in embryos. Therefore, the rise in *BnACBP2* expression during seed development and the overall high level of *BnACBP6* expression throughout this period mean that the two *BnACBPs* encoded by these genes are together likely involved in oil accumulation.

Hills et�al. (1994) first reported that *BnACBP6* was more highly expressed in *B. napus* developing embryos and cotyledons than in leaves and flowers. The RNA-Seq results reported here, as well as other studies on embryos/seeds of major oil accumulating plants, indicate that *BnACBP6* is the most highly expressed of all the *BnACBPs* (Troncoso-Ponce et�al. 2011, Chen et�al. 2015). Furthermore, *BnACBP6* expression gradually decreased during seed development, consistent with the microarray data reported by Troncoso-Ponce et�al. (2011). A similar trend for *ACBP6* expression has also been reported for *Ricinus communis* endosperm, *Euonymus alatus* endosperm and Arabidopsis seeds (Troncoso-Ponce et�al. 2011). BnACBP6 protein declined to low levels by the later stages of seed development (Brown et�al. 1998) in agreement with our data showing that *BnACBP6* expression significantly decreased from early to later stages of development.

AtACBP6 from Arabidopsis was shown to bind long-chain acyl-CoA (C16 to C18-CoA) esters at least as efficiently as other Arabidopsis ACBPs such as ACBP4 and ACBP5 (Hsiao et�al. 2015a). In addition, recombinant AtACBP6 (rAtACBP6) binds to long-chain acyl-CoA esters with *K*_d_ values ranging from 36 to 84 nM, in comparison to rAtACBP4 with ranges of 3–190 �M and rAtACBP5 with ranges of 35–92 �M (Hsiao et�al. 2015a). Furthermore, Arabidopsis *acbp6* mutant embryos accumulated more 18:1-CoA (Hsiao et�al. 2015a). Transgenic Arabidopsis developing seeds overexpressing *BnACBP6* showed reduced 18:1-CoA because the overexpressed BnACBP6 could bind more 18:1-CoA (Yurchenko et�al. 2014). These results confirm a role for ACBP6 in acyl-CoA transport in seeds. Furthermore, seed weight was reduced in Arabidopsis double (*acbp4*, *acbp6* and *acbp5*, *acbp6*) and triple (*acbp4*, *acbp5*, *acbp6*) mutants, further implying a significant role for ACBP6 in seed development (Hsiao et�al. 2015a).

An extra dimension to the data is provided by our analysis of *BnACBP* isoforms that arise via alternative pre-mRNA splicing. *BnACBP6* has four isoforms, the majority (*BnACBP6-1*, *BnACBP6-2* and *BnACBP6-3*) declined significantly in the period 27–38 DAF and then decreased further toward the end of the seed maturation (45 DAF). By contrast, *BnACBP6-4* expression increased at the end of the development. Even though the major *BnACBP6* isoforms declined during development, they were still the most abundant *ACBP* transcripts at 38 DAF which is near the end of the rapid phase of oil accumulation, and at 45 DAF. The increase in both *BnACBP2* isoform transcripts during development suggests that both play a similar role in lipid biosynthesis. Indeed, it can be noted that although AtACBP6 is a small molecular mass protein confined to the cytosol (Chen et�al. 2008), AtACBP2, an ankyrin-repeat protein, is associated with the ER (Li and Chye 2003). Thus, we would suggest that these two ACBPs play complementary roles during oil accumulation. BnACBP6 (and its homologs) would be mainly involved in cytosolic acyl-CoA binding and inter-organelle transport, whereas BnACBP2 (and its homologs) would be important for enzyme interactions within the Kennedy pathway for TAG biosynthesis. The significance of ACBPs in the transport of acyl-CoA esters during embryo development has also been reported in mice where the depletion of the ACBP6 homolog as well as the phosphotyrosine-binding-domain-containing ACBD3 led to embryonic lethality (Zhou et�al. 2007, Landrock et�al. 2010).

### *ACBP* expression in the seed coat contrasts with that in the embryo

Several *BnACBP* genes were significantly expressed in seed coats at 27 DAF. *BnACBP1*, *BnACBP2*, *BnACBP3* and *BnACBP4* all showed moderate expression levels that were higher than in embryos except for *BnACBP1*, whereas *BnACBP5* had the lowest expression level. Nevertheless, as in embryos, *BnACBP6* was the most highly expressed of all *BnACBP* genes. However, in contrast to the embryos, the level of *BnACBP6* increased in the seed coat during seed development. As in embryos, *BnACBP2* increased steadily so that, by 45 DAF, its level was about three times that at 27 DAF. Although both *BnACBP1* and *BnACBP4* showed small decreases during seed development, *BnACBP3* gave a peak of expression in the rapid oil accumulation phase at 38 DAF. Thus, most *BnACBPs* showed significant expression during this period pointing to complementary roles within the seed coat. In a sense, this is to be expected, because the embryo’s main function is to produce and store oil, while the seed coat has to ensure germination and seedling establishment. The myriad of BnACBPs available for these purposes includes significant amounts of five of the six classes which have membrane-localized or soluble features (Du et�al. 2016). It has been reported previously that AtACBP1 and AtACBP2 have been immunolocalized to the developing embryo using antibodies specific to AtACBP1 and AtACBP2, respectively, and both displayed expression in developing seeds coinciding with lipid deposition (Chye et�al. 1999, Chen et�al. 2010). Furthermore, AtACBP1 has been detected to the plasma membrane of heart-, torpedo- and cotyledonary-staged embryos as well as in the seed coat as revealed by immunoelectron microscopy (Chye et�al. 1999). Several of the ACBPs, such as AtACBP1 (Du et�al. 2013, Lung et�al. 2017, Chen et�al. 2018, Lung et�al. 2018) and AtACBP2 (Gao et�al. 2009, Gao et�al. 2010), have been proven to possess the potential to interact with other proteins based on the presence of the characteristic Class II ankyrin-repeat domain or the Class IV Kelch domain (Du et�al. 2016). Within the BnACBP classes in seed coats, most isoforms behaved similarly in terms of their expression. The exception was *BnACBP6* where *BnACBP6-1* declined while *BnACBP6-2* and *BnACBP6-3* increased and *BnACBP6-4* remained constant during development. Thus, comparative data for embryos and seed coat emphasize the subtle differences between spatially adjacent tissues where their physiology is distinct.

We used a combination of RNA-Seq and qRT-PCR for measurement of gene expression levels and found that gene expression values were generally in very good agreement, particularly for the more highly expressed *BnACBPs.* However, sometimes while the trend in expression level change was similar, the magnitude of differential expression varied between the two methods, which may be attributable to their different sensitivities. For the lower expressed *BnACBPs*, such as *BnACBP3* and *BnACBP5* and some of the low abundance isoforms, there were small deviations between RNA-Seq and qRT-PCR data but this likely reflects the limitations in quantifying very low gene expression levels. We realize that changes in expression levels of *ACBP* genes may not necessarily translate directly to alterations in metabolism (Voelckel et�al. 2017). Moreover, in our past research on oil accumulation in oilseed rape, we have identified several factors other than ACBP levels that influence the process (e.g. Perry et�al. 1999, Weselake et�al. 2008, Tang et�al. 2012, Woodfield et�al. 2018). Nevertheless, the data reported here identify which ACBPs may be important for oil accumulation in embryos or lipid metabolism in seed coats.

In conclusion, this study has provided comprehensive information on differentially expressed lipid-related genes between embryos and seed coats and *ACBP* (isoform) expression in an important oil crop, *B. napus*. The apparent importance for BnACBP2 and BnACBP6 and their isoforms during oil accumulation offers possibilities for genetic manipulation which may significantly enhance TAG formation. In a world with limited agricultural land but increasing demand for vegetable oils, this is a very important potential application.

## Materials and Methods

### Plant materials

Wild-type *B. napus* cv. DH12075 (LEAR) seeds were germinated in pots containing soil mix (Tref Substrates, Jiffy). Ten-day-old seedlings were transplanted individually into 8.7-inch pots. Plants were grown in a greenhouse with a temperature of approximately 23�C and with a natural light period (11–13 h) at the School of Biological Sciences, the University of Hong Kong. Flowers were pollinated manually and tagged on the first day when flowers open. Siliques were collected at 27, 38 and 45 DAF, representing early, rapid and late stages of lipid accumulation in the oilseed rape plants.

### Morphological analysis of Hong Kong-grown *Brassica* embryos

Due to the differences in temperature, light intensity and daylight length between the greenhouses in Hong Kong (11–13 h of natural light) and Cardiff (16 h with a light intensity of 250 �mol�m^−2^�s^−1^; Woodfield et�al. 2017, Woodfield et al. 2018), wild-type *B. napus* grew slower in Hong Kong than Cardiff. For Cardiff-grown wild-type *B. napus*, siliques were collected at 20, 27 and 35 DAF, representing early, rapid and late stages of lipid accumulation (Turnham and Northcote 1983, Woodfield et�al. 2017, Woodfield et al. 2018). To match these with those of Hong Kong-grown *B. napus*, the morphology of embryos at different developmental stages from Hong Kong-grown wild-type *B. napus* was compared with the embryo architecture described by Borisjuk et�al. (2013). Results were confirmed by lipid analysis. Subsequently, 27, 38 and 45 DAF were selected to represent early, rapid and late stages of lipid accumulation in silique collection. Embryos were dissected manually by a razor blade and photographed (Woodfield et�al. 2017). Seeds from 10 siliques from each of the six plants were harvested for measurements of fresh weight.

### FA profiling

Twelve seeds from different siliques were harvested at 27, 38 and 45 DAF for each biological repeat. In total, six biological repeats were used. FA extraction was performed as previously described (Woodfield et�al. 2017, Woodfield et al. 2018). Seed samples were incubated in 1.2 ml of isopropanol at 70�C for 30 min to inactivate any endogenous (phospho-) lipases. Nonadecanoic acid (19:0; Sigma, St. Louis, Missouri, USA) was used as an internal standard. Fatty acid methyl esters (FAMEs) were analyzed by an Agilent GC-MS device (5,973 inert mass spectrometer combined with 6,890 N gas chromatograph) equipped with an Agilent J&W DA-WAX capillary column (30 m � 0.25 mm � 0.25 �m; Lung et�al. 2017). The oven temperature was set to 170�C for 3 min, increased to 220�C at 4�C�min^−1^, and held at 220�C for 15 min (Woodfield et�al. 2017). FAMEs were routinely identified by comparing the retention time of peaks with the Supelco 37 Component FAME MIX standard (Sigma) but had been identified fully in previous work (Woodfield et�al. 2017).

### RNA extraction

Siliques were harvested at 27, 38 and 45 DAF. Embryo and seed coat samples were dissected manually by a razor blade and stored immediately in liquid nitrogen before RNA extraction. Total RNA from embryos was extracted using an RNeasy Plant Mini kit (Qiagen, Hilden, Germany). Total RNA from seed coats which contain procyanidins was extracted according to Wang and Vodkin (1994) with some modifications. Seed coat samples were first ground to a fine powder in liquid nitrogen, an equal volume of special RNA extraction buffer (450 �l) [100 mM Tris-HCl, pH 8.0, 20 mM EDTA-Na, 1.5% SDS (w/v), 200 mM NaCl, 5% bovine serum albumin (BSA; w/v), 4% polyvinylpyrrolidone (w/v)] and the lysis buffer (RLC; 450 �l) from RNeasy Plant Mini Kit with 1% 2-mercaptobenzothiazole (v/v) were added and mixed by vortex. Then 21.2 �l of proteinase K (10 mg/ml) was added and incubated at 37�C with gentle shaking (80 rpm) for 20 min to digest the remaining BSA. Subsequently, seed coat RNA was extracted following the instructions of the RNeasy Plant Mini Kit (Qiagen). On-column DNase digestion (Qiagen) was performed on RNA samples to remove potential DNA contamination.

### RNA-Seq analysis

Embryo and seed coat RNA samples from each of the three developmental stages, with three biological replicates, were sent to Beijing Genomics Institute (BGI, Hong Kong, China) for RNA-Seq analysis (BGISEQ-500; Mak et�al. 2017). The *B. napus* genome from ‘Darmor-*bzh*’ was used as a reference genome (Chalhoub et�al. 2014). The gene expression level was quantified using a software package (RSEM) (Li and Dewey 2011). The FPKM values which represent expression levels for each transcript were generated (Li and Dewey 2011). The NoISeq method (Tarazona et�al. 2015) was used to screen for DEGs with the following criteria: fold change ≥2 (log2 ratio ≥1) and diverge probability ≥0.8. GO enrichment analysis was performed by using the Cluster and Java Treeview software. GO classification was conducted using the WEGO software (Ye et�al. 2006). Subsequently, pathway enrichment analysis of DEGs was carried out using the KEGG database (Kanehisa et�al. 2008).

### Quantitative RT-PCR

Total RNA (2.5 �g) was reverse-transcribed using the Transcriptor First Strand cDNA Synthesis Kit (Roche, Mannheim, Germany) following the manufacturer’s instructions. Subsequently, qRT-PCR was performed with the FastStart Universal SYBR Green Master (Roche) on the StepOnePlus Real-Time PCR System (Applied Biosystems) under the following conditions: 95�C for 10 min, followed by 40 cycles of 95�C for 15 s and 60�C for 30 s. Three independent biological repeats were performed for each reaction, and three technical repeats were conducted in each PCR reaction. *Brassica TIP41-lIKE PROTEIN* (*TIP41*) was used as internal control (see Chen et�al. 2010). The data were analyzed using the 2^−ΔΔCt^ method (Schmittgen and Livak 2008). Each *BnACBP* primer was designed from sequences originating from the conserved region of *BnACBP* isoforms within the same class. Each *BnACBP* isoform-specific primer was designed from a specific region for each isoform. Details of primers for qRT-PCR are listed in Supplementary Table S3.

## Funding

A Royal Society grant (IE 160011), Biotechnology and Biological Sciences Research Council (BBSRC) [Grant numbers BB/M02850X/1 and BB/L009420/1], the Wilson and Amelia Wong Endowment Fund, Research Grants Council of Hong Kong [AoE/M-05/12], Innovation Technology Fund of Innovation Technology Commission: Funding Support to State Key Laboratories in Hong Kong and HKU Committee on Research and Conference Grants (CRCG) award [1611159027]. P.L. was supported by a Postdoctoral Fellowship from AoE/M-05/12 and the University of Hong Kong.

## Supplementary Material

pcz169_Supplementary_Figures-Tables
