# *Erratum*Comparative Transcriptomics Analysis of *Brassica napus* L. during Seed Maturation Reveals Dynamic Changes in Gene Expression between Embryos and Seed Coats and Distinct Expression Profiles of Acyl-CoA-Binding Proteins for Lipid Accumulation

**DOI:** 10.1093/pcp/pcz220

**Published:** 2019-12-31

**Authors:** Pan Liao, Helen K Woodfield, John L Harwood, Mee-Len Chye, Simon Scofield

*Plant and Cell Physiology,* https://doi.org/10.1093/pcp/pcz169Published: 28 August 2019

In the original published version of the article, there were two typos; one in the abstract, and one on page 8, which affected the scientific clarity of the research. This has now been corrected.

