# Supplementary material for: Comparative Transcriptomics Analysis of Brassica napus L. during Seed Maturation Reveals Dynamic Changes in Gene Expression between Embryos and Seed Coats and Distinct Expression Profiles of Acyl-CoA-Binding Proteins for Lipid Accumulation
Source: Plant Cell Physiol. 2019 Aug 28;60(12):2812–25. doi: 10.1093/pcp/pcz169 (PMC6896696; doi:10.1093/pcp/pcz169)
Supplement: pcz169_Supplementary_Figures-Tables [file pcz169_supplementary_figures-tables.pdf]

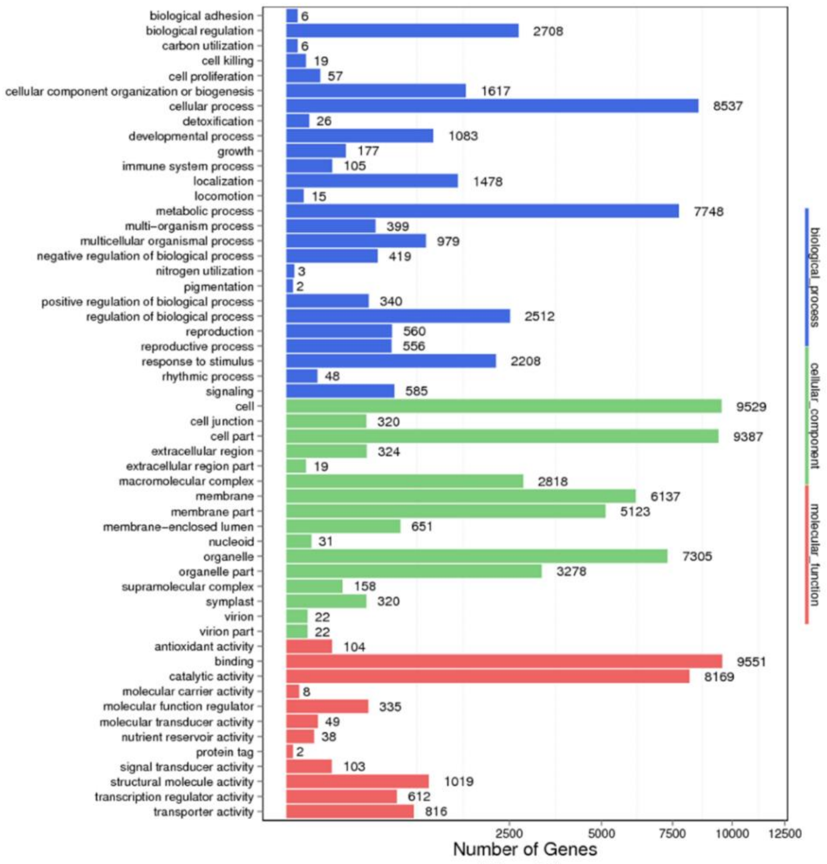

27 vs 38

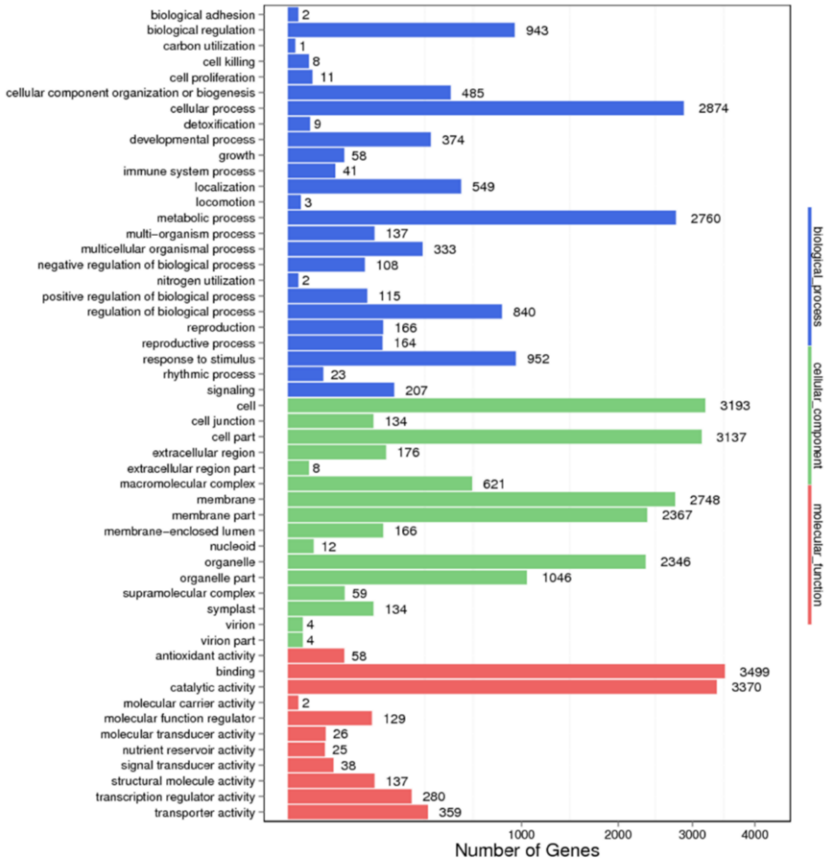

38 vs 45

DAF EM

Supplementary Figure S1. Gene ontology (GO) classification for DEGs identified in 27 vs 38 DAF and 38 vs 45 DAF comparisons for embryos (EM).

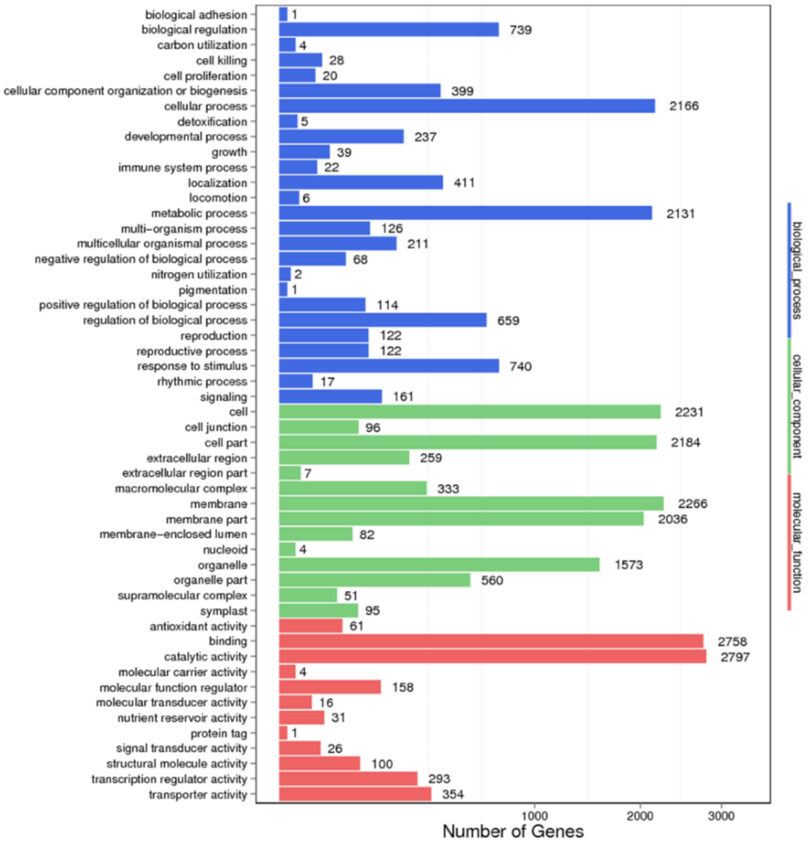

27 vs 38
DAF SC

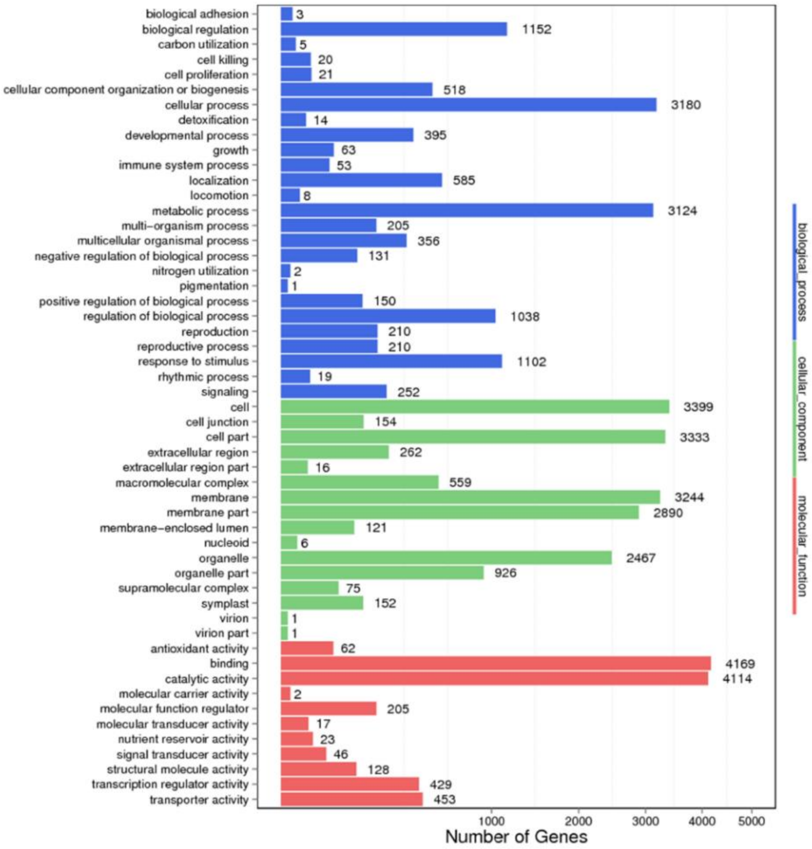

38 vs 45
DAF SC

Supplementary Figure S2. Gene ontology (GO) classification for DEGs identified in 27 vs 38 DAF and 38 vs 45 DAF comparisons for seed coats (SC).

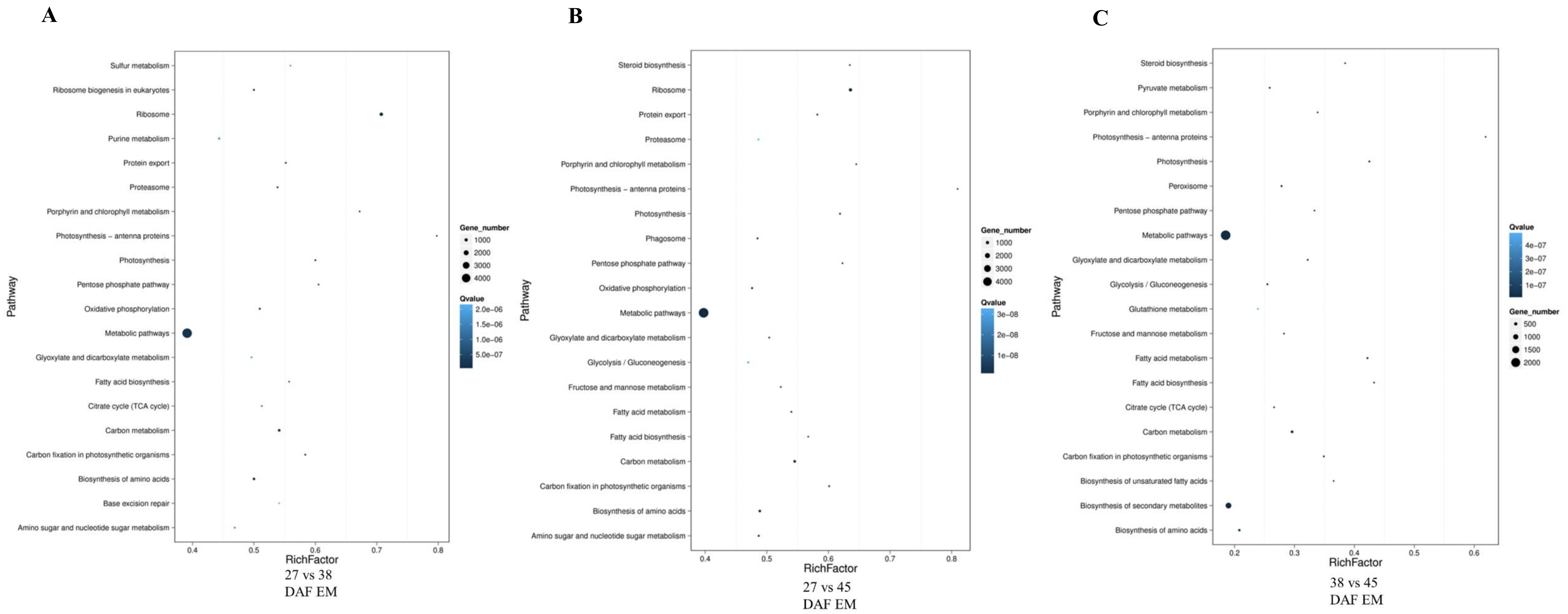

Supplementray Figure S3. KEGG pathway enrichment for DEGs for each pairwise in *B. napus* embryos (EM). (A) 27 vs 38 DAF. (B) 27 vs 45 DAF. (C) 38 vs 45 DAF.

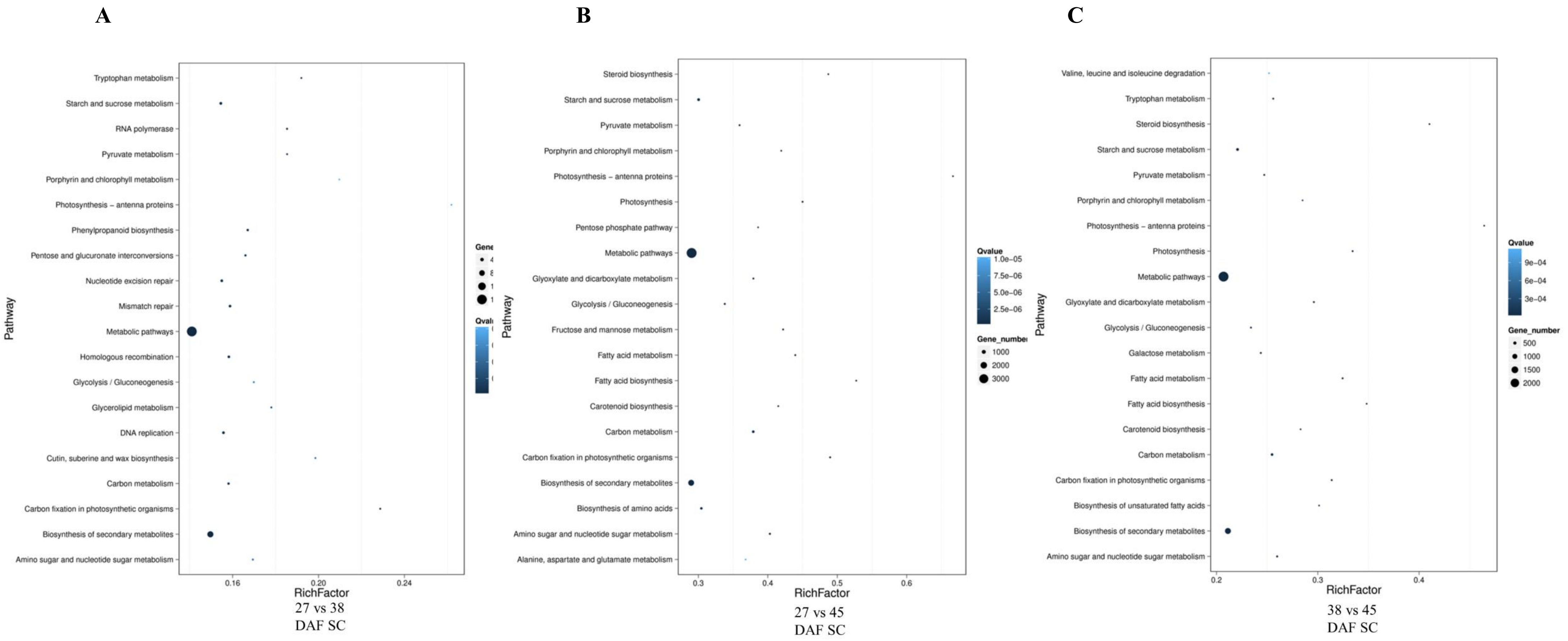

Supplementary Figure S4. KEGG pathway enrichment for DEGs for each pairwise in *B. napus* seed coats (SC). (A) 27 vs 38 DAF. (B) 27 vs 45 DAF. (C) 38 vs 45 DAF.

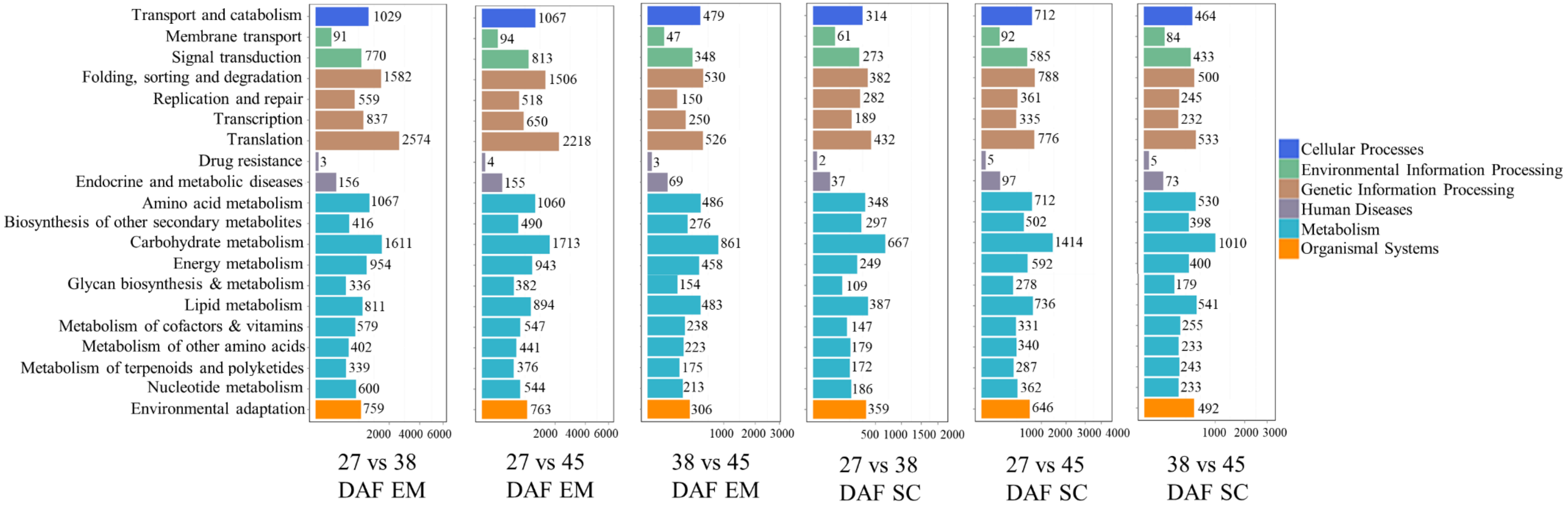

Supplementary Figure S5. KEGG second pathway terms for each pairwise comparison in embryos (EM) and seed coats (SC).

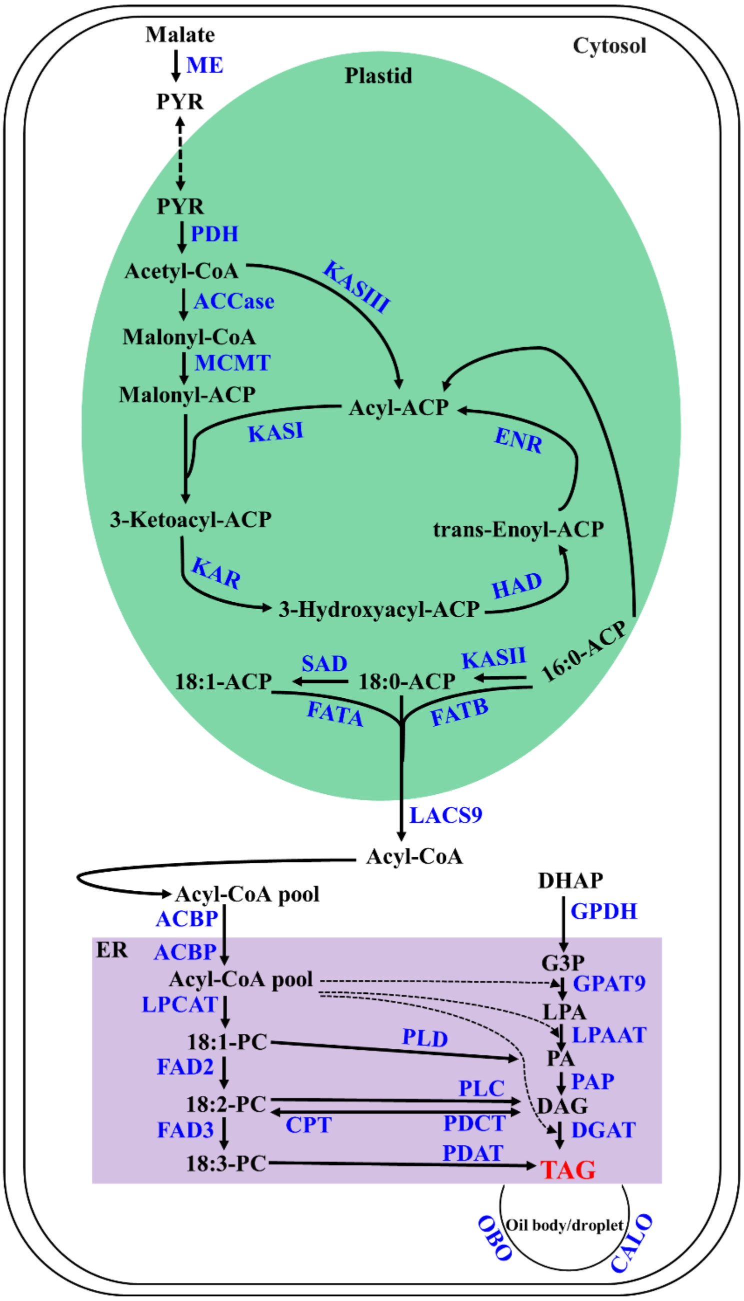

Supplementary Figure S6. General overview of fatty acid and triacylglycerol (TAG) biosynthesis in maturing seeds of *Brassica napus*. Fatty acid (FA) biosynthesis occurs in the plastid (green), while FA elongation and TAG assembly occur at the endoplasmic reticulum (ER) (purple). TAG is highlighted in red. The synthesised TAG will be packaged into oil body/ lipid droplets for storage. Dihydroxyacetone phosphate (DHAP) can come from glycolysis. Cytosol- and ER-localised acyl-CoA-binding proteins (ACBPs) co-exist in plants. Phospholipid:diacylglycerol acyltransferase (PDAT), phospholipase C (PLC) and phospholipase D (PLD) can use substrates other than those indicated in the diagram. Enzymes

involved are shown in blue. Additional abbreviations: ACCase, acetyl-CoA carboxylase; ACP, acyl carrier protein; CALO, caleosin; CPT, CDP-choline:diacylglycerol cholinephosphotransferase; DGAT, diacylglycerol acyltransferase; ENR, enoyl-ACP reductase; FAD, fatty acid desaturase; FATA and FATB, fatty acyl-ACP thioesterases; GPAT, glycerol 3-phosphate acyltransferase; GPDH, glycerol-3-phosphate dehydrogenase; HAD, β-hydroxyacyl-ACP dehydratase; KAR, ketoacyl-ACP reductase; KAS, ketoacyl-ACP synthase; LACS, long-chain acyl-CoA synthetase; LPAAT, lysophosphatidic acid acyltransferase; LPCAT, lysophosphatidylcholine acyltransferase; ME, malic enzyme; MCMT, malonyl-CoA:ACP malonyltransferase; OBO, oil body oleosin; PAP, phosphatidic acid phosphatase; PC, phosphatidylcholine; PDH, pyruvate dehydrogenase; PDCT, phosphatidylcholine:diacylglycerol cholinephosphotransferase; PYR, pyruvate; SAD, stearoyl-ACP desaturases.

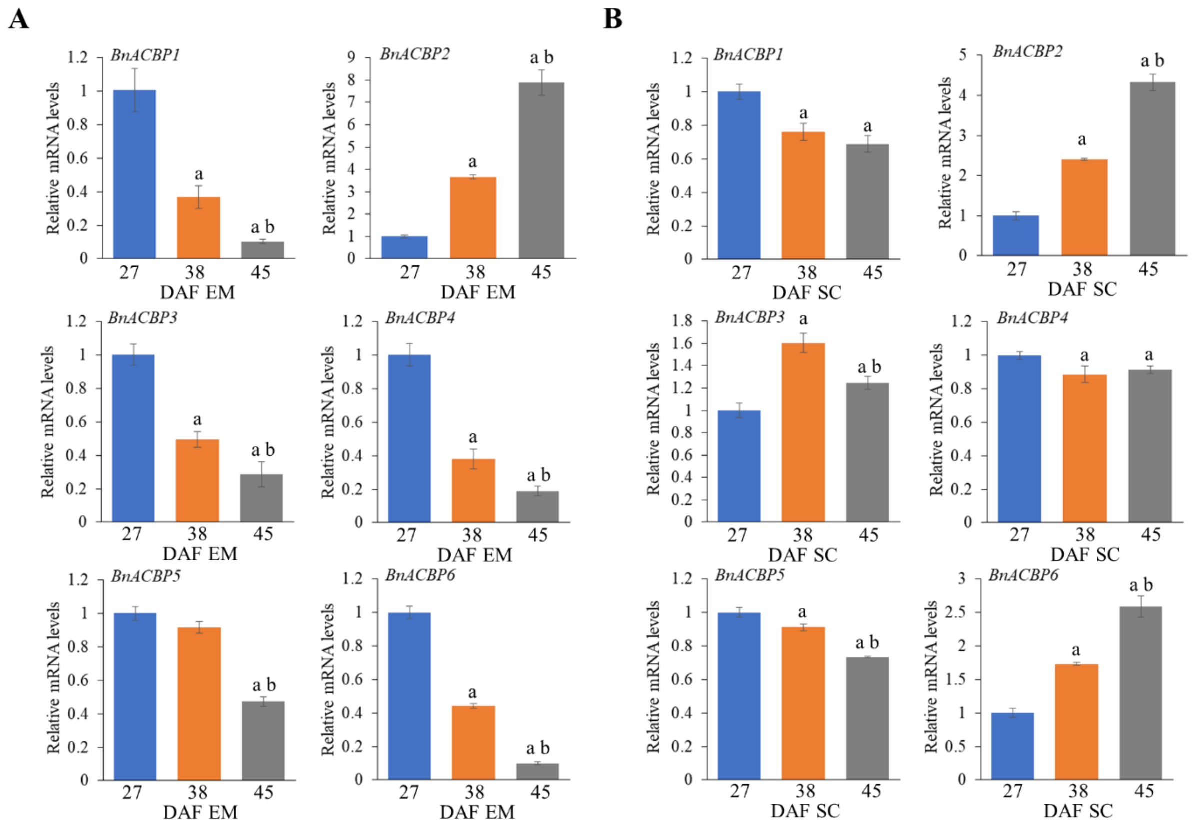

Supplementary Figure S7. Expression profiling of *B. napus ACBPs* in embryos (A) and seed coats (B) during seed development by qRT-PCR. Total RNA was extracted from dissected embryos at 27, 38, and 45 DAF. Expression levels relative to 27 DAF were normalised to expression of *TIP41*. Data are means ± SD of three independent replicates. a = significant difference ($P < 0.05$ by Student's t-test) when 38 DAF or 45 DAF compared with 27 DAF; b = significant difference ($P < 0.05$ by Student's t-test) between 38 DAF and 45 DAF.

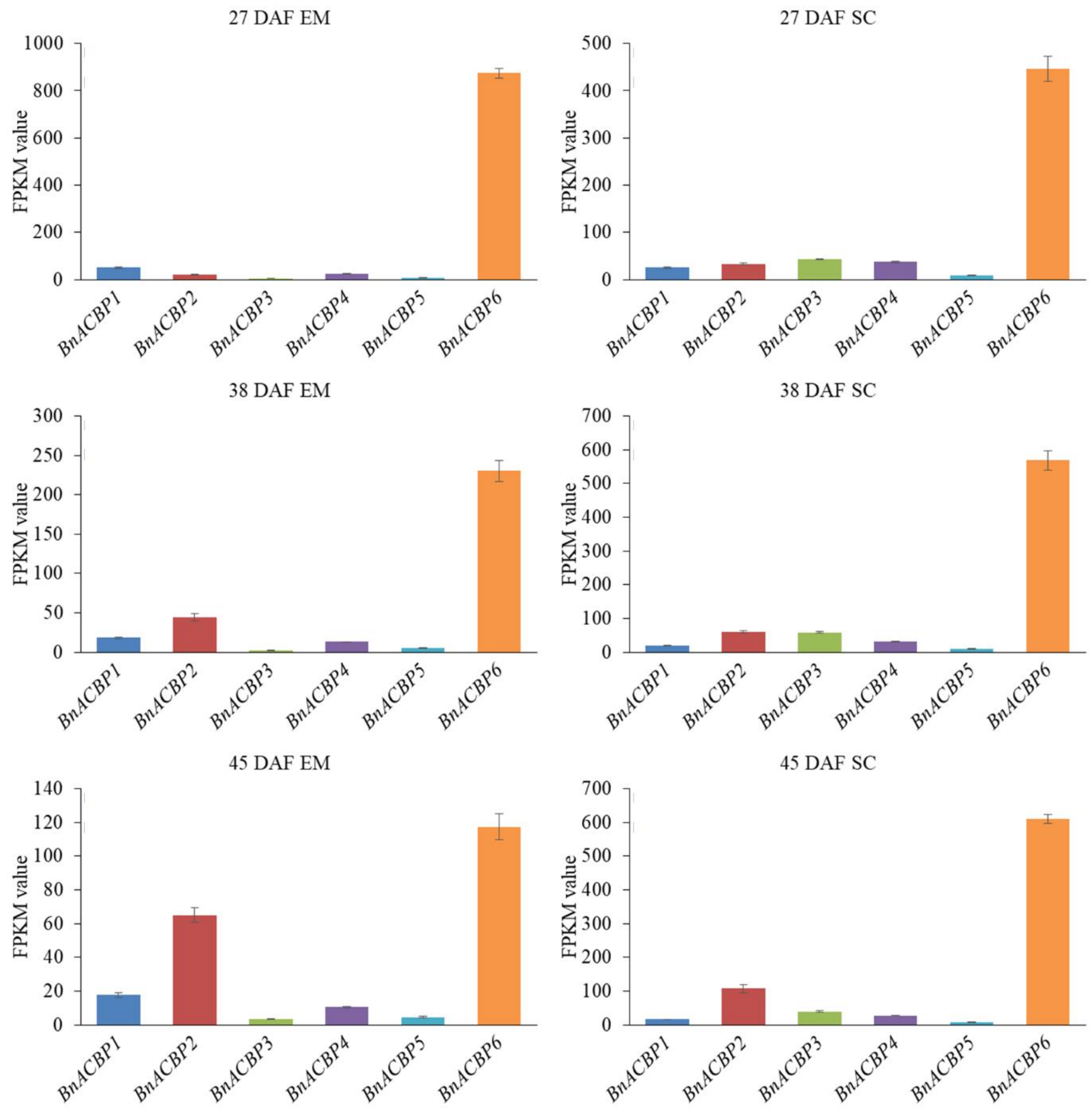

Supplementary Figure S8. Quantification of BnACBP transcript levels 27 DAF, 38 DAF and 45 DAF embryo (EM) and seed coat (SC) samples by RNA-seq. Average fragments per kilobase of transcript per million fragments mapped (FPKM) values from three biological repeats were used to calculate transcript (expression) values.

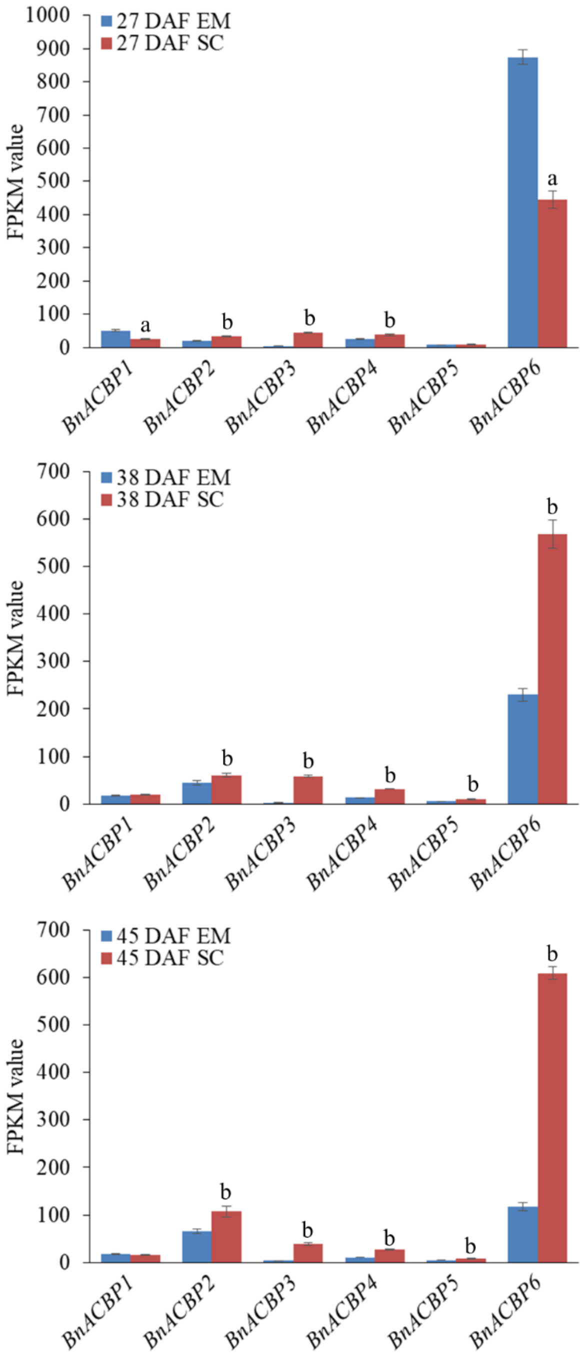

Supplementary Figure S9. Comparison of BnACBP expression in embryos (EM) and seed coats (SC) at 27 DAF, 38 DAF and 45 DAF by RNA-seq. Average fragments per kilobase of transcript per million fragments mapped (FPKM) values from three biological repeats were used to calculate transcript (expression) values.

Supplementary Table S1. Changes in the percentage of major fatty acid (FA) species, total FA content (µg mg FW^{-1} or µg per seed) and seed fresh weight (mg) during oil accumulation in oilseed rape

	27 DAF	38 DAF	45 DAF
16:0	10.0 ± 0.21	**5.0 ± 0.03[a]**	**4.8 ± 0.24[a]**
16:1 n7	1.3 ± 0.01	**0.6 ± 0.02[a]**	**0.6 ± 0.04[a]**
18:0	4.1 ± 0.01	**2.9 ± 0.01[a]**	**1.4 ± 0.22 [a,b]**
18:1 n9	38.0 ± 0.15	**65.8 ± 0.32[a]**	**66.3 ± 2.37[a]**
18:1 n7	6.9 ± 0.10	**3.2 ± 0.03[a]**	**2.9 ± 0.09[a]**
18:2	27.0 ± 0.01	**12.3 ± 0.14[a]**	**13.8 ± 1.36[a]**
18:3	10.2 ± 0.03	**6.5 ± 0.07[a]**	**7.2 ± 0.61[a]**
20:0	1.3 ± 0.01	**1.4 ± 0.02[a]**	**1.0 ± 0.03[a,b]**
20:1	0.6 ± 0.01	**1.3 ± 0.02[a]**	**1.3 ± 0.01[a]**
Total FA (µg mg FW^{-1})	21.1 ± 0.75	**99.9 ± 2.08[a]**	**112.9 ± 2.71[a,b]**
Total FA (µg per seed)	115.6 ± 4.12	**657.5 ± 58.02[a]**	**806.1 ± 19.38[a,b]**
One seed FW (mg)	5.9 ± 0.55	**7.2 ± 0.23[a]**	**8.0 ± 0.26[a,b]**

Seeds of wild-type *B. napus* cv. DH12075 (LEAR) were collected 27, 38, and 45 days after flowering (DAF), representing early, rapid and late stages of oil accumulation in *B. napus*. Fatty acid (FA) were analysed by GC-MS. Values are shown by means ± s.d. (n=6). a, significant difference ($P < 0.05$ by Student's *t*-test) when 38 DAF or 45 DAF compared with 27 DAF; b, significant difference ($P < 0.05$ by Student's *t*-test) between 38 DAF and 45 DAF. Values displaying significant changes are shown in bold. Values increased in comparison to control are marked in red, values decreased in comparison to control are marked blue. Total FA were calculated on the basis of fresh weight (FW) or of each fresh seed relative to the internal control (19:0 FA). Major FA species (> 0.5% of total) are listed above.

Supplementary Table S2. Core differentially expressed gene numbers for DEGs detected in 27 vs 38, 27 vs 45 and 38 vs 45 comparisons for embryos (top) and seed coats (botom).

In EM, 5673 DEGs commonly found in three comparisons	No. of upregulated DEGs	No. of downregulated DEGs
27 vs 38	769	4904
27 vs 45	888	4785
38 vs 45	1350	4323

In SC, 3197 DEGs commonly found in three comparisons	No. of upregulated DEGs	No. of downregulated DEGs
27 vs 38	1672	1525
27 vs 45	1633	1564
38 vs 45	1423	1774

Supplementary Table S3. Oligonucleotide primers used for qRT-PCR in this study.

Primer name	Gene name	Accession number	Orientation	Sequence (5'-3')
ML2954	*BnACBP1*	BnaA02g10270D, BnaC02g44810D	Forward	TTGAGTGAAGCGGCGAAAAC
ML2955			Reverse	GGCGAGAAGGTAAGCGAAG
ML2956	*BnACBP2*	BnaA01g16660D, BnaC01g20440D	Forward	CATTGAAAGCGGCATTCCTGT
ML2957			Reverse	AGTTTGGCCTTCGTTGTCCT
ML2958	*BnACBP3*	BnaA01g13710D, BnaA03g46540D, BnaC01g16110D, BnaC07g38820D	Forward	TGCTTCTCACGGCGGTT
ML2959			Reverse	GACCACCGGAGCATCCA
ML2960	*BnACBP4*	BnaA03g29000D (AIS76194), BnaA05g31780D (AIS76195/AIS7620), BnaAnng02420D (AIS76196), BnaC03g34240D (AIS76199), AIS76201	Forward	TGGTGGATACAATGGACGTTACA
ML2961			Reverse	GCAGAGAGACTAACTGGCAAAGG
ML2962	*BnACBP5*	BnaC02g39790D (AIS76197), BnaA02g31230D (AIS76198)	Forward	GAGCGCTACCCTCGCTTAC
ML2963			Reverse	AGTTGTTTCACGGAAGACGGT
ML2964	*BnACBP6*	BnaAnng25690D, BnaA05g36060D, BnaCnng15340D, BnaA08G07670D	Forward	AGGAGCACGCTGAGAAAGTCA
ML2965			Reverse	TCCCAGGACGACTGGTGG
ML3265	*BnACBP1-1*	BnaA02g10270D	Forward	TGCTTCTGTAACGGTTTTAGGC

ML3266			Reverse	CAAGGCAAACTAGTCTCTAGCTG
ML3267	*BnACBP1-2*	BnaC02g44810D	Forward	TTGTCTTTTGAGTGAAGCGGC
ML3268			Reverse	TCAAACAACAGCGAAGGAGGA
ML3269	*BnACBP2-1*	BnaA01g16660D	Forward	GCCGCATCGAGTCTTCTACC
ML3270			Reverse	TGTCACCAGCGACGCTATC
ML3271	*BnACBP2-2*	BnaC01g20440D	Forward	TGGATGTTTCAGAGTCTTGTCCT
ML3272			Reverse	TGTTACCGGCAAAGTCGGTT
ML3015	*BnACBP3-1*	BnaA01g13710D	Forward	AACCATTGGGTGAGGATTAGGA
ML3016			Reverse	AGAATCCTTTGGGAAAATAAAGGC A
ML3017	*BnACBP3-2*	BnaA03g46540D	Forward	GCTCTTTGGGAATACAAAGGCA
ML3018			Reverse	TGTTTTTGTTTGCTGCAACTCC
ML3281	*BnACBP3-3*	BnaC01g16110D	Forward	TGCTAGCTTCAAATCCAACTCT
ML3282			Reverse	TCTTGTTCAAGCCTTTCCTGC
ML3287	*BnACBP3-4*	BnaC07g38820D	Forward	ACCTTAGGCACAACTGGTGTT
ML3288			Reverse	GGTGACGGTGACCCAAAGAG
ML3285	*BnACBP4-1*	BnaA03g29000D (AIS76194)	Forward	TAGCTTCTCCGTCTCCGATCA
ML3286			Reverse	GCCAGATGTGGCCCTAGTTA
ML3275	*BnACBP4-2*	BnaC03g34240D (AIS76199)	Forward	ACGAACTCTAAACGACAGAGC
ML3276			Reverse	TCATCTTTCTCCTGAGGGCT
ML3277	*BnACBP4-3*	BnaA05g31780D (AIS76195/AIS76200)	Forward	TCTTCCCGTATACTCGCTCCA
ML3278			Reverse	AAACCATGCGTGAGACTCGG
ML3279	*BnACBP4-4*	BnaAnng02420D (AIS76196)	Forward	CTAAACGTCAGAGCTCCGGT
ML3280			Reverse	ATGGCGAATCATCATCCTTCT
ML3023	*BnACBP5-1*	BnaC02g39790D (AIS76197)	Forward	AACTTGAACCCGACGGTTGT
ML3024			Reverse	CTGCTTCGCCGTATTAGCCA
ML3291	*BnACBP5-2*	BnaA02g31230D (AIS76198)	Forward	TGTCTCTTCCATTACCAGCCTC
ML3292			Reverse	ACCAACTCTGACAAGTGTACCA
ML2986	*BnACBP6-1*	BnaAnng25690D	Forward	TGATTCACCAAATGCTCCACA

ML2987			Reverse	GCGTCAAGAATCTTCGTTTAGACC
ML2988	*BnACBP6-2*	BnaA05g36060D	Forward	GCAAAATTGAAGTGATGTGTGC
ML2989			Reverse	TGTGGTTTAACTGTTTATAGAGGTG
ML2990	*BnACBP6-3*	BnaCnng15340D	Forward	ATTTGAGCCAGAGAGCCTCG
ML2991			Reverse	AGCGTAAGACGGAGAAGACG
ML2992	*BnACBP6-4*	BnaA08g07670D	Forward	TCCTCTGTCTGCAGTAGTCCT
ML3077			Reverse	ACCAAGACCAAGTTCACAAAGT
ML3073	*TIP41*	EV222761	Forward	AGAGTCATGCCAAGTTCATGGTT
ML3074			Reverse	CCTCATAAGCACACCATCAACTCTAA
